# The role of clathrin in post‐Golgi secretion in plant cells

**DOI:** 10.1111/nph.71454

**Published:** 2026-07-20

**Authors:** Maciek Adamowski, Adam Gackowski, Ivana Matijević, Saqer S. Alotaibi, Jiří Friml

**Affiliations:** ^1^ Institute of Science and Technology Austria Am Campus 1 3400 Klosterneuburg Austria; ^2^ Intercollegiate Faculty of Biotechnology University of Gdańsk and Medical University of Gdańsk Abrahama 58 80‐307 Gdańsk Poland; ^3^ Department of Biotechnology, College of Science Taif University PO Box 11099 Taif 21944 Saudi Arabia

**Keywords:** auxilin, AUXILIN‐LIKE, clathrin, endocytosis, exocytosis, secretion, *trans*‐Golgi Network

## Abstract

- Within the plant endomembrane system, the vesicle coat protein clathrin localizes to the plasma membrane (PM) and the *trans‐*Golgi Network/early endosome (TGN/EE). While the role of clathrin in endocytosis at the PM is well established, its function at TGN/EE, presumably in late secretion (trafficking from the TGN/EE to the cell surface) or en route to the vacuole, is debated. Similarly debated are potential homeostatic mechanisms balancing the trafficking routes, especially endocytosis and late secretion.
- We address these questions in *Arabidopsis thaliana* using conditional silencing of *CLATHRIN HEAVY CHAIN* (*CHC*), conditional overexpression of the clathrin uncoating factor AUXILIN‐LIKE1, and secretory mutants.
- *CHC* silencing interferes with trafficking of cargoes destined for the apoplast and the PM, supporting a function of clathrin in late secretion. The secretory cargoes become abnormally rerouted from the TGN/EE to the vacuole. Unlike *CHC* silencing, overexpression of AUXILIN‐LIKE1 selectively inhibits clathrin‐mediated endocytosis while secretion continues normally at early points of induction. Conversely, secretory mutants exhibit a reduced PM recruitment of clathrin, and variably, of the TPLATE endocytic component.
- Together, our data show a role of clathrin in secretion and suggest secretion as a fundamental trafficking process to which endocytosis is adjusted by a weak homeostatic mechanism.

## Graphical abstract

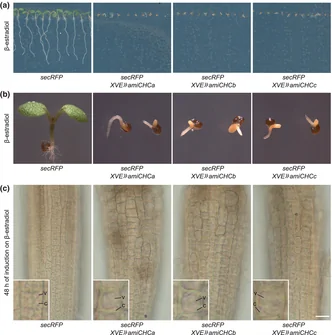

## Introduction

Vesicles coated with the coat protein clathrin are the main carriers responsible for endocytosis in plant cells (Dhonukshe *et al*., 2007; Kitakura *et al*., 2011; Larson *et al*., 2017). The mechanism of clathrin‐mediated endocytosis (CME) in plants is an active field of study, revealing both conserved and plant‐specific features of the clathrin‐coated vesicle (CCV) formation machinery (Narasimhan *et al*., 2020; Dahhan & Bednarek, 2022; Kraus *et al*., 2024; Adamowski *et al*., 2024a). Beside the plasma membrane (PM), where CME occurs, clathrin is also present at the *trans*‐Golgi Network/early endosome (TGN/EE; Ito *et al*., 2012; Kang *et al*., 2011). The function of clathrin at this compartment is usually assigned to the formation of CCVs with cargoes destined for the vacuolar route (Robinson & Pimpl, 2014; Buchanan *et al*., 2015). However, TGN/EE is also the final intracellular compartment of the conventional secretory pathway leading from the endoplasmic reticulum (ER), through the Golgi apparatus (GA) and TGN, to the PM (Žarský *et al*., 2009; Wang *et al*., 2017). The final vesicular transport step from the TGN to the PM occurs by secretory vesicles (SV) thought to form from the TGN without protein coats (Žarský *et al*., 2009). Yet, indirect lines of evidence link clathrin to the late secretory process. At the TGN, CCVs are formed with the aid of Adaptor Protein 1 (AP‐1) complex (Owen *et al*., 2004; Park *et al*., 2013; Wang *et al*., 2013; Robinson & Pimpl, 2014). Super‐resolution microscopy indicates a distinct domain on the plant TGN enriched in both AP‐1 and clathrin and defined by the secretory R‐SNARE (SNAP RECEPTOR) VAMP721, supporting clathrin's role in post‐Golgi secretion (Shimizu *et al*., 2021). Loss‐of‐function mutants of *AP1M2*, encoding an AP‐1 subunit, exhibit deficiencies in secretion of soluble proteins (Park *et al*., 2013), while mutants in *AP1/2β* are deficient in post‐Golgi secretion in the tapetum (Liu *et al*., 2022). The AP‐1 complex is recruited to membranes as an effector of activated ARF small GTPases (Paczkowski *et al*., 2015), and inhibition of BIG ARF‐GEFs (Guanine nucleotide Exchange Factors for ARFs), the TGN‐localized ARF activators, leads to an arrest of secretion like *ap‐1* loss of function, concomitant with loss of AP‐1 recruitment to membranes (Richter *et al*., 2014). Together, these results are consistent with a scenario where AP‐1‐containing CCVs formed with the aid of ARFs activated by BIG ARF‐GEFs participate in late secretion from the TGN. Yet, *ap1m2* mutants exhibit defects of vacuolar traffic as well (Park *et al*., 2013), and an AP‐1‐binding motif mediating vacuolar targeting of cargo has been described (Wang *et al*., 2014). Additionally, TGN‐localized monomeric clathrin adaptors MODIFIED TRANSPORT TO THE VACUOLE1 (MTV1) and EPSIN1 indicate clathrin functions in both vacuolar and secretory pathways (Sauer *et al*., 2013; Collins *et al*., 2020; Heinze *et al*., 2020). Finally, evidence from loss‐of‐function mutants of the *Arabidopsis thaliana* AP‐3 adaptor complex are too, indirectly suggestive of clathrin's function in the vacuolar pathway (Feraru *et al*., 2010; Zwiewka *et al*., 2011), although in contrast with the association of AP‐3 with CLATHRIN HEAVY CHAIN (CHC) detected by immunoprecipitation (Zwiewka *et al*., 2011), AP‐3 was not enriched in purified plant CCVs, suggesting its clathrin‐independent function (Dahhan *et al*., 2022). Taken together, the accumulated data warrant a closer investigation into the function of TGN‐localized clathrin.

Cargo transport in vesicles leads to the movement of the membrane material, from which the compartments themselves are made, within the endomembrane system. Thus, it is conceivable that to correctly maintain the individual compartments, the rates of individual trafficking processes must be mutually coordinated. For instance, at the PM, the amount of membrane incoming by exocytosis and being removed by endocytosis might require to be mutually adjusted to accommodate for cell growth while retaining optimal membrane tension. The question of so understood ‘trafficking homeostasis’ has been a matter of some research. For instance, some yeast secretory (*sec*) mutants (Novick *et al*., 1980; Spang, 2015), especially those deficient in its late stages, are defective in endocytosis (Riezman, 1985). The yeast Rab GTPase Sec4p has been described as a secretory component triggering endocytic events at the PM, coupling the two processes (Johansen *et al*., 2016). PM turnover in plant cells as a result of secretion and endocytosis was reviewed as early as 1988 (Steer, 1988), while the processes that may require endo‐ and exocytic coupling were discussed recently (Zhang *et al*., 2019). Interestingly, Larson *et al*. (2017) report that *clathrin heavy chain1* (*chc1*) mutants are defective in secretion of transiently expressed secretory yellow fluorescent protein (secYFP), while *syntaxin of plants 121* (*syp121*), deficient in a Qa‐SNARE required for exocytosis, exhibits reduced internalization of an endocytic marker dye FM4‐64. A recent study further supports a mutual regulation between post‐Golgi secretion and CME, on the basis of reduced rates of traffic and membrane recruitment of components in mutants of the reciprocal pathway (Yan *et al*., 2021). Shifts in the relative rates of endocytosis and exocytosis in response to changes in osmotic conditions were described as well (Zwiewka *et al*., 2015). Together, these results are indicative of mechanisms for mutual regulation between secretion and endocytosis. Yet, inhibition of CME by overexpression of the putative CCV uncoating factor AUXILIN‐LIKE1 causes an excess accumulation of membrane at the cell periphery that likely results from a continued secretory activity (Adamowski *et al*., 2018). This observation argues against a strong homeostatic coupling of the two processes. Furthermore, it is worth noting that certain membrane lipid types are known to be transported at membrane contact sites between ER and *trans*‐Golgi/TGN, as well as ER and PM, in a nonvesicular manner that employs lipid‐transfer proteins (Holthuis & Menon, 2014; Saheki & De Camilli, 2017; Mesmin *et al*., 2019). This mode of lipid transport represents a potential alternative mechanism for maintaining homeostasis in the endomembrane system without the need to coordinate vesicular trafficking pathways.

In the present work, we explore these two linked questions: that of a potential function of clathrin in late secretion, and that of a potential mutual regulatory relationship between secretion and endocytosis, in particular, CME. We make use of lines expressing inducible artificial microRNA (amiRNA) targeting *CHC*, which we compare with the deleterious effects of overexpression of AUXILIN‐LIKE1 (Adamowski *et al*., 2018). We also analyse CME in several secretory mutants with the use of confocal laser scanning microscopy (CLSM) and total internal reflection fluorescence (TIRF) microscopy on fluorescent reporters for endocytic clathrin‐coated pits (CCPs). Our findings reveal a role of clathrin in the secretory pathway and suggest a weak homeostatic mechanism adjusting endocytosis to the rates of secretion, but not vice versa.

## Materials and Methods

### Plant material

The following previously described *Arabidopsis thaliana* (L.) Heynh. lines were used in this study: *35S*_*pro*_:*secRFP* (Samalova *et al*., 2006; NASC ID N799370), *CLC2*_*pro*_:*CLC2‐GFP* (Konopka *et al*., 2008), *CLC2*_*pro*_:*CLC2‐GFP UBQ10*_*pro*_:*mCh‐AUXILIN‐LIKE1* (Adamowski *et al*., 2018), *LAT52*_*pro*_:*TPLATE‐GFP RPS5A*_*pro*_:*AP2A1‐TagRFP tplate* (Gadeyne *et al*., 2014), *DRP1C*_*pro*_:*DRP1C‐GFP* (Konopka *et al*., 2008), *GNOM*_*pro*_:*GNOM‐LIKE1‐GFP* (Adamowski *et al*., 2024b), *BEN3*_*pro*_:*BEN3‐TagRFP* (Kitakura *et al*., 2017), *PIN1*_*pro*_:*PIN1‐GFP* (Benková *et al*., 2003), *XVE»AUXILIN‐LIKE1* (Adamowski *et al*., 2018), *ap1m2–1* (Park *et al*., 2013), *echidna* (Gendre *et al*., 2011), and *sec5a‐2 sec5b‐1* (Zhu *et al*., 2018). Lines generated as part of this study are listed in Supporting Information Table S1 and primers used for genotyping in Table S2.

### *In vitro* cultures of *Arabidopsis* seedlings

Seedlings were grown in *in vitro* cultures on ½‐strength Murashige and Skoog (½MS) of pH = 5.9 supplemented with 1% (w/v) sucrose and 0.8% (w/v) phytoagar at in 16 h : 8 h, 21°C : 21°C, light : dark cycles with Philips GreenPower LED as light source, using deep red (660 nm)/far red (720 nm)/blue (455 nm) combination, with a photon density of *c*. 140 μmol/(m^2^s) ± 20%. Beta‐estradiol (Sigma‐Aldrich, St. Louis, MO, USA) was solubilized in 100% ethanol to 5 mg ml^−1^ stock concentration and added to ½MS during preparation of solid media to a final concentration of 2.5 μg ml^−1^. Seedlings of *XVE»amiCHC* and *XVE»AUXILIN‐LIKE1* lines were induced by transferring to beta‐estradiol‐supplemented media at Day 3 or 4. Heat‐shock induction of *HS*_*pro*_:*TMK4‐GFP* and *HS*_*pro*_:*PIN1‐GFP* was initiated 7–8 h before live imaging and carried out for 1 h at 37°C.

### Light microscopy

Images of *XVE»amiCHC* seedlings developing in *in vitro* cultures were taken with a Leica EZ4 HD stereomicroscope, and microscopic images of root apical meristems (RAMs) were taken with an Olympus BX53 light microscope.

### Epifluorescent microscopy

Images of TPLATE‐GFP and AP2A1‐TagRFP signals in root tips of *XVE»amiCHCa* line were captured using a Nikon Eclipse TE300 microscope. Signal levels in regions of interest encompassing entire RAMs were measured in Fiji (https://imagej.net/Fiji) as mean grey values. Background signal was measured in each image and subtracted from the measured fluorescent protein signal.

### Confocal Laser Scanning Microscopy

Four‐ to five‐day‐old seedlings were used for live imaging with Zeiss LSM800 confocal laser scanning microscope with 20× 0.8 air and 40× 1.2 immersion objectives and Leica TCS SP8 X confocal laser scanning microscope with 20× 0.75 immersion objective. For comparative studies of TMK4‐GFP and PIN1‐GFP localizations in *XVE»amiCHCa* and *XVE»AUXILIN‐LIKE1* lines, z‐stacks of nine images at 1 μm intervals were captured. Quantifications were performed using Fiji (https://imagej.net/Fiji). Total signal intensities of CLC2‐GFP were measured as mean grey value of entire RAMs visible in CLSM images. PM signal intensities of TPLATE‐GFP and AP2A1‐TagRFP in *XVE»amiCHCa*, as well as PIN1‐GFP, were measured as mean grey values of a line of 5‐pixel width drawn over multiple PMs in each CLSM image. Cell plate signal intensities were measured similarly with 7 pixel‐wide lines. PM signal intensities of TPLATE‐GFP in secretory mutants were measured as mean grey values of a line of 4‐pixel width drawn over multiple PMs in each CLSM image and normalized to mean grey values of entire RAMs visible in the images. GNL1‐GFP and BEN3‐TagRFP compartments were counted in 60 × 60 pixel regions encompassing cytoplasm, corresponding to 79.772 μm^2^ (GNL1‐GFP) or 105.508 μm^2^ (BEN3‐TagRFP).

### Total Internal Reflection Fluorescence microscopy

Early elongation zone of roots in excised *c*. 1 cm long root tip fragments from 7‐d‐old seedlings were used for TIRF imaging. Imaging was performed with Olympus IX83 TIRF microscope, using a 100× TIRF lens with an additional 1.6× or 2× magnification lens in the optical path. Time lapses of 100 frames at 1‐s intervals with exposure times of 200 ms, or single snapshots of 200 ms exposure, were taken, depending on the experiment. Quantifications were performed using Fiji (https://imagej.net/Fiji). PM foci of DRP1C‐GFP, CLC2‐GFP, and TPLATE‐GFP were counted in square regions of 36 μm^2^ taken from the captured TIRF images or movies. TGN signals of CLC2‐GFP were excluded from quantification based on size distinction. Lifetimes of CLC2‐GFP and TPLATE‐GFP were measured in stacks of kymographs generated from time lapses using Reslice function. Approximately 200 vesicle formation events (tracks) per time lapse were measured. Any tracks that were clearly distinguishable and were captured in their entirety in the time lapse were taken into consideration. Diameters of five representative GNL1‐GFP and BEN3‐TagRFP compartments were measured per image.

### Immunostaining

BFA (B7651; Sigma‐Aldrich) was solubilized in dimethyl sulfoxide to 50 mM stock concentration and added to liquid ½MS for treatments. Immunostaining was performed on 4‐d‐old seedlings according to a previously published automated protocol (Sauer *et al*., 2006). CHC was stained with rabbit anti‐CHC antibody (AS10 690, 1 : 600; Agrisera, Vännäs, Sweden). As secondary antibodies, Goat anti‐Rabbit conjugated with Alexa Fluor 488 (A11034, 1 : 600; Invitrogen, Carlsbad, CA, USA) was used for CHC co‐localization with secRFP, and Goat anti‐Rabbit conjugated with Cy5 (A10523, 1 : 600; Invitrogen) was used for co‐localization of CHC with PIN1‐GFP and TMK4‐GFP.

### FM4‐64 staining

For colocalization of PIN1‐GFP and TMK4‐GFP with membrane filaments, seedlings were stained with 1 μM FM4‐64 (T13320; Invitrogen) in liquid ½MS supplemented with 1% (w/v) sucrose in darkness for 5 min, washed to remove excess dye, and mounted on microscopy slides for live imaging. Line plots of signal colocalization were generated using the Plot Profile function in Fiji (https://imagej.net/Fiji). For FM4‐64 uptake measurements, seedlings were stained with 4 μM FM4‐64 in liquid ½MS medium supplemented with 1% (w/v) sucrose in darkness for 5 min on ice, washed to remove excess dye on ice, and mounted on microscopy slides at room temperature, which marked the starting point of internalization time. Quantifications were performed in Fiji as a ratio between mean grey values of a region of interest containing cell interiors of a representative cell file and a region of interest consisting of 5 pixel‐wide lines drawn over membranes of the corresponding cells. Background signal was measured in each image and subtracted from both measurements before calculating the ratio.

### Neutral Red staining

Seedlings were stained in 2 μM Neutral Red (229 810 250; Thermo Fisher, Waltham, MA, USA) in ½MS of pH = 5.9 supplemented with 1% (w/v) sucrose at room temperature for 40 min, washed twice for 1 min, and mounted on microscopy slides for live imaging. Seedlings evaluated at time point 0 h after heat shock or room temperature control were stained first, returned onto a solid medium for temperature treatments, and imaged directly after temperature treatments to avoid the delay caused by 40‐min staining.

### Molecular cloning

All constructs generated in this study are listed in Table S3 and primers used for cloning in Table S2. Artificial microRNA sequences targeting *CHC* genes were designed using the Web MicroRNA Designer website (http://wmd3.weigelworld.org/). The following amiRNA sequences were employed: amiCHCa TCTCGCAGTACTTACCCACAA; amiCHCb TGCAAAATTTACTGCTCCCTG; amiCHCc TTATTTGACCAGTCTTGGCAG. amiRNA constructs were cloned by overlap extension PCR with iProof High Fidelity polymerase (Bio‐Rad, Hercules, CA, USA), following a protocol published on the Web MicroRNA Designer website. The amiRNA sequences were introduced into pDONR221 Gateway entry vectors (Invitrogen) by BP Clonase and subsequently combined by LR Clonase II with UBQ10‐promoter driven XVE elements from p1R4‐pUBQ10:XVE (Siligato *et al*., 2016) in pH7m24GW,3, pK7m24GW,3 (*amiCHCa*) and pB7m24GW,3 (*amiCHCa*,*b*,*c*) expression vectors (Karimi *et al*., 2002). TMK4 and PIN1‐GFP‐2 coding sequences were cloned into pDONR221 Gateway entry vectors by BP Clonase. Heat‐shock‐inducible expression vectors were generated by LR Clonase II with proHS/pDONRP4P1r and GFP/pDONRP2rP3 (for TMK4‐GFP) in expression vectors pK7m34GW (TMK4‐GFP) and pK7m24GW,3 (PIN1‐GFP‐2).

### Quantitative reverse transcriptase PCR

Total RNA was isolated from 5‐d‐old seedlings using RNeasy Plant Mini Kit (Qiagen, Hulsterweg 82, 5912 PL, Venlo, The Netherlands). cDNA was synthesized using iScript™ cDNA Synthesis Kit (Bio‐Rad). qPCR was performed with Luna® reagent (New England Biolabs, Ipswich, MA, USA). Two primer pairs detecting both *CHC1* and *CHC2* transcripts were employed. *TUB2* and *PP2AA3* were used as reference genes and relative *CHC*, *TPLATE‐GFP*, and *AP2A1‐TagRFP* expression was quantified with ΔCT method adapted for the use of multiple reference genes (Riedel *et al*., 2014) with modifications. Primer sequences are listed in Table S2.

### CHC protein level evaluation

Total protein was isolated from 5‐d‐old seedlings of *secRFP XVE»amiCHCa* using lysis buffer (50 mM Tris pH 7.6, 150 mM NaCl, 1% Triton X‐100, cOmplete™ Protease Inhibitor Cocktail (Roche, Grenzacherstrasse 124, 4070 Basel, Switzerland)). Equal amount of protein from each sample was loaded onto 4–15% Mini‐PROTEAN® TGX Stain‐Free™ Protein Gels (Bio‐Rad) for electrophoresis before semi‐dry transfer onto low fluorescence (LF) polyvinylidene fluoride (PVDF) membranes (*Trans*‐Blot Turbo RTA Transfer Kit; Bio‐Rad). Total protein was visualized on membranes by Stain‐Free technology as loading control. Membranes were blocked in 3% nonfat milk in Tris‐buffered saline with Tween 20 (TBST), probed with 1 : 2000 anti‐CHC antibody (AS10 690; Agrisera), washed, and probed with 1 : 10000 anti‐rabbit horseradish peroxidase (HRP)‐conjugated antibody (NA9340; Cytiva, Marlborough, MA, USA). Signals were detected using SuperSignal™ West Pico PLUS Chemiluminescent Substrate (Thermo Scientific, Waltham, MA, USA). Mean intensity of regions of interest encompassing CHC bands was measured in ImageJ and background signal subtracted. Measured CHC levels were corrected for gel loading through dividing by mean intensity of total protein in lanes as visualized by Stain‐Free technology, with background signal subtracted. Loading‐corrected CHC levels from induced samples (mean of two biological replicates) were divided by mean value of mock samples to calculate protein depletion.

## Results

### Generation of inducible lines silencing *CHC*

To address directly the function of clathrin in secretion, we generated amiRNA lines targeting genes encoding CHC, which together with CLATHRIN LIGHT CHAIN (CLC) comprise the vesicular clathrin coat. We cloned three different amiRNA sequences, named *amiCHCa*, *amiCHCb*, and *amiCHCc*, all of which target both *CHC* homologues in *A. thaliana*, *CHC1* and *CHC2*. We expressed the amiRNAs in stably transformed *A. thaliana* plants under the control of the β‐estradiol‐inducible expression system. *XVE»amiCHCa*, *XVE»amiCHCb*, and *XVE»amiCHCc* constructs were introduced into the *secRFP* marker line, which expresses an RFP variant inserted into the ER and secreted to the apoplast through the secretory pathway (Samalova *et al*., 2006).

Downregulation of *CHC1* and *CHC2* expression after 48 h of amiRNA induction was confirmed by qPCR in lines expressing each amiRNA variant (Fig. S1A). CHC protein levels were reduced approximately twofold after 48 h of amiRNA induction, while only a modest reduction in CHC levels was observed at earlier time points of 16 h, 24 h, and 32 h, as verified by western blotting in a representative *XVE»amiCHCa* line (Fig. S1B).

**Fig. 1:**
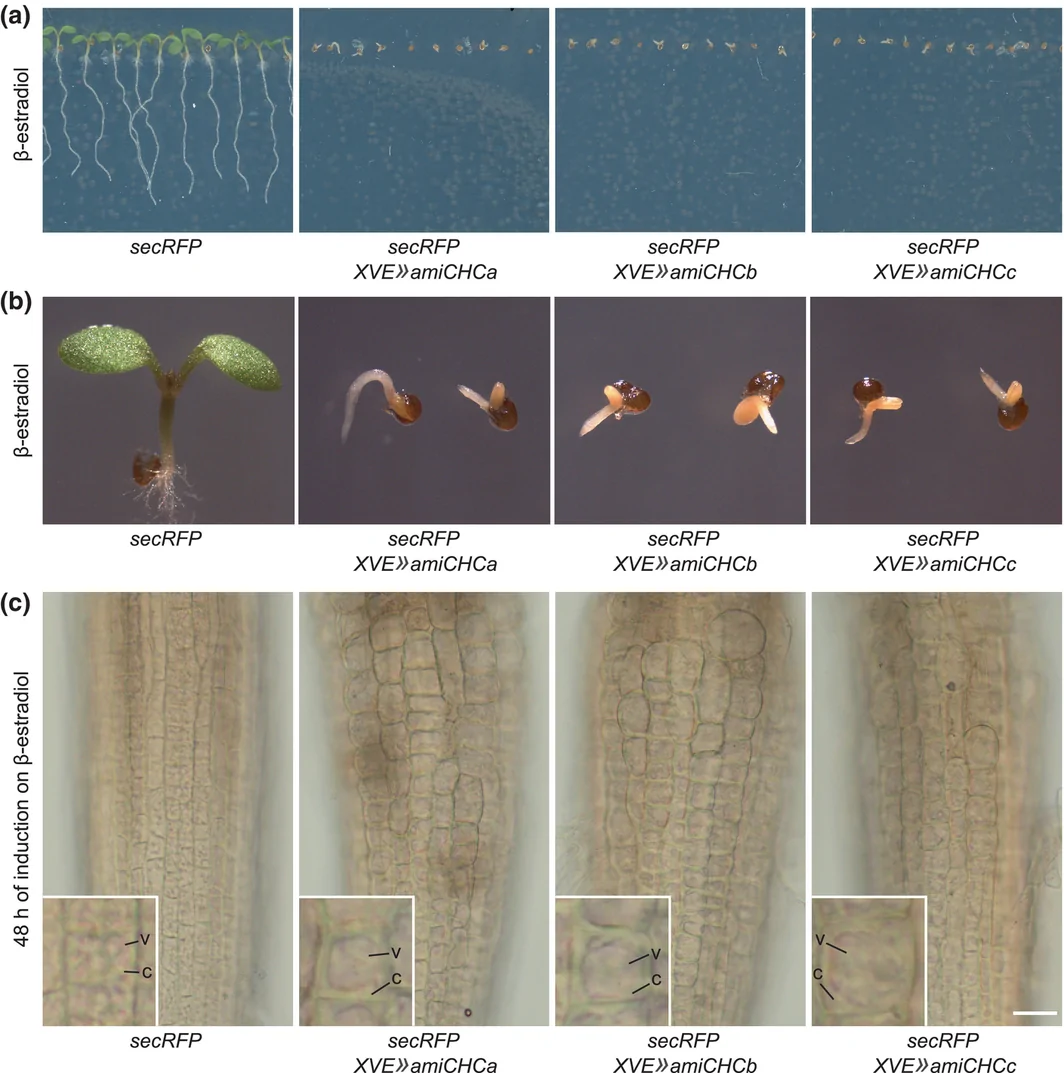
Morphology of *Arabidopsis thaliana XVE»amiCHC* seedlings. (a) *secRFP XVE»amiCHC* lines grown on a medium supplemented with β‐estradiol for 6 d. Silencing of *CLATHRIN HEAVY CHAIN* (*CHC*) leads to a severe delay in development. Mock controls in Supporting Information Fig. S2A. (b) Detail of seedlings shown in (a). (c) Microscopic images of root apical meristems (RAMs) of *secRFP XVE»amiCHC* lines after 48 h of induction by β‐estradiol. The RAMs of *XVE»amiCHC* lines exhibit enlarged cells with swollen vacuoles. Insets show magnified cells with a large, central vacuole (V) surrounded by a thin layer of cytoplasm (C) in *XVE»amiCHC* lines, compared with fragmented vacuoles in *secRFP*. Mock controls in Fig. S2B. Bar, 10 μm.

When cultured *in vitro* on media supplemented with β‐estradiol, *XVE»amiCHCa*, *XVE»amiCHCb*, and *XVE»amiCHCc* seedlings exhibited greatly diminished rates of growth and development. After 6 d of growth, the seedlings were of minute size, usually without appreciable outgrowth of the root, with small cotyledons without green pigmentation (Fig. 1a,b, S2A). When seedlings of these lines were germinated on standard media, transferred onto media supplemented with β‐estradiol at Day 3, and their RAMs observed under a light microscope 48 h later, the cells in the RAM were found to be enlarged, and characterized by large, swollen vacuoles (Fig. 1c, S2B). This morphological characteristic was similar to that observed previously in lines inducibly overexpressing AUXILIN‐LIKE1/2, leading to arrest of CME (Adamowski *et al*., 2018).

In summary, interference with clathrin function by inducible silencing of *CHC* led to a severe inhibition of growth and development. In the following, using *amiCHCa*, we first characterize the impact of *CHC* silencing on CME and on internal endomembrane compartments, and then, its impact on secretion.

### Disruption of CME by *CHC* silencing

To assess the effect of *CHC* silencing on CME and thus verify the correct function of the amiRNA transgene, we introduced *XVE»amiCHCa* into marker lines expressing fluorescent protein fusions of several main components of CME. We analysed CLATHRIN LIGHT CHAIN2 (CLC2; Konopka *et al*., 2008), an isoform of CLC; TPLATE, a subunit of the TPLATE complex, participating in plant CME presumably as an adaptor protein complex (Gadeyne *et al*., 2014); AP2A1, a subunit of the AP‐2 adaptor complex (Di Rubbo *et al*., 2013); and DYNAMIN RELATED PROTEIN 1C (DRP1C; Konopka *et al*., 2008), a plant‐specific isoform of dynamin, a mechanoenzyme acting in the separation of nascent CCV from the PM. Elsewhere, we similarly scrutinized SH3 DOMAIN‐CONTAINING PROTEIN2 (SH3P2), a late component of nascent CCVs (Adamowski *et al*., 2024a). In this and following sections, *amiCHC* constructs were induced for *c*. 48 h before fluorescent live imaging unless otherwise specified.

**Fig. 2:**
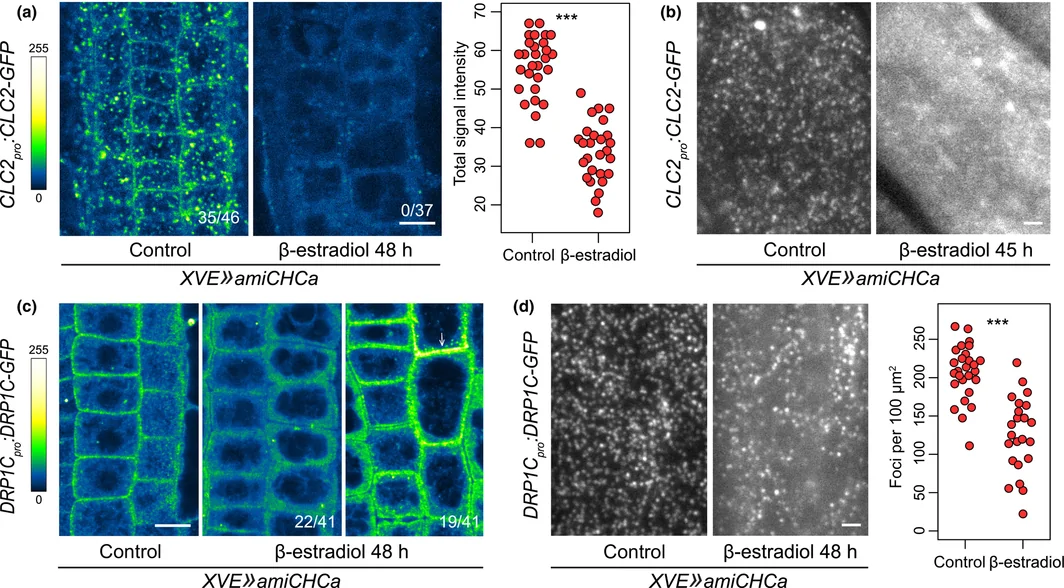
Effects of *CLATHRIN HEAVY CHAIN* (*CHC*) silencing in *Arabidopsis thaliana XVE»amiCHCa* on CLC2‐GFP and DRP1C‐GFP. (a) Confocal laser scanning microscopy (CLSM) images of CLC2‐GFP in root apical meristems (RAMs) of control and induced seedlings of *XVE»amiCHCa*. Downregulation of *CHC* lead to loss of plasma membrane (PM) signals, as well as the majority of *trans‐*Golgi Network/early endosome (TGN/EE) signals, and a reduction in total CLC2‐GFP signal levels. Numbers in bottom right corner indicate the ratio of roots with PM signals. Graph shows quantification of total signal intensity; each data point represents a single root. Control: 56 ± 8 a.u. (mean ± SD), *n* = 29; β‐estradiol: 34 ± 8 a.u., *n* = 27. Values were compared using a *t*‐test, ***, *P* ≤ 0.001. Bar, 10 μm, h, hours. (b) Total internal reflection fluorescence (TIRF) images of CLC2‐GFP in roots of control and induced seedlings of *XVE»amiCHCa*. Following silencing of *CHC*, CLC2‐GFP is observed as a bright diffuse signal in the cytosol, with few clathrin‐coated pits (CCPs) and TGN‐localized CLC2‐GFP signals visible in some of the samples. Bar, 2 μm, h, hours. (c) CLSM images of DRP1C‐GFP in RAMs of control and induced seedlings of *XVE»amiCHCa*. Downregulation of *CHC* lead to variable outcomes, with 22/41 seedlings exhibiting loss of PM localization of DRP1C‐GFP (middle panel), and 19/41 retaining PM signals of normal or abnormally high intensity (right panel, arrow). Bar, 10 μm, h, hours. (d) TIRF images of DRP1C‐GFP in roots of control and induced seedlings of *XVE»amiCHCa*. Downregulation of *CHC* caused a reduction in average density of DRP1C‐positive foci at the PM. Graph shows collated quantifications of average foci density from all experiments, each data point represents a single root. Mock: 207 ± 35 foci per 100 μm^2^ (mean ± SD), *n* = 27; β‐estradiol: 125 ± 48 foci per 100 μm^2^, *n* = 23. Values were compared using a *t*‐test, ***, *P* ≤ 0.001. Bar, 2 μm, h, hours.

When assessed by CLSM in seedling RAM epidermis, silencing of *CHC* led to a loss of CLC2‐GFP signals from the PM in all seedlings analysed (Fig. 2a). CLC2‐GFP was also lost from the majority of TGN/EE compartments, with only scarce TGN/EE signals remaining (Fig. 2a, compare with Fig. 4b, see later). These effects were concomitant with a general decrease in CLC2‐GFP signal levels (Fig. 2a). Consistently, when assessed by TIRF microscopy in early elongation zones of the root, in the wild‐type CLC2‐GFP appeared as dense foci representing CCPs on the PM, as well as larger objects representing TGN compartments, while following *CHC* silencing, only scarce PM‐localized CCPs, as well as TGN signals, could be seen (Fig. 2b; Videos S1, S2). In summary, downregulation of *CHC* decreased the abundance of CLC and caused loss of most CLC from its sites of action, the PM and TGN.

The effects of *CHC* silencing on DRP1C‐GFP observed with CLSM in RAM epidermis were variable. The PM signal of DRP1C‐GFP was decreased or absent in approximately half of the seedlings (Fig. 2c, middle) while in the other half of the analysed population, the signals either remained normal or were clearly increased compared with the control (Fig. 2c, right, arrow). TIRF microscopy in early elongation zones of the root indicated an approximately twofold decrease in the average density of DRP1C‐positive foci at the PM following downregulation of *CHC* (Fig. 2d), while cells with abnormally high densities of DRP1C‐positive foci were not observed.

**Fig. 3:**
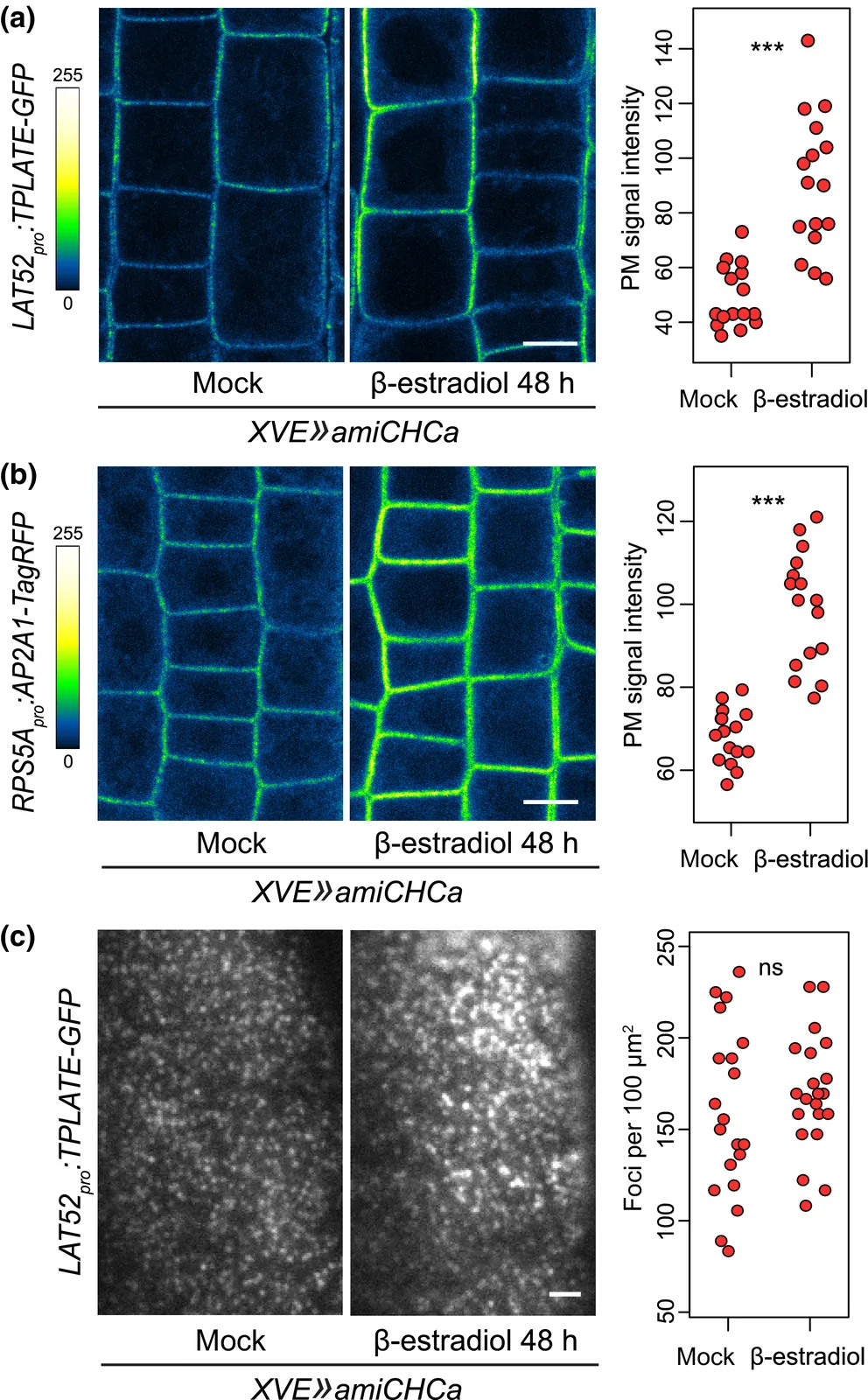
Effects of *CLATHRIN HEAVY CHAIN* (*CHC*) silencing in *Arabidopsis thaliana XVE»amiCHCa* on TPLATE‐GFP and AP2A1‐TagRFP. Confocal laser scanning microscopy (CLSM) images of TPLATE‐GFP (a) and AP2A1‐TagRFP (b) in root apical meristems (RAMs) of control and induced seedlings of *XVE»amiCHCa*. Downregulation of *CHC* lead to an increase in plasma membrane (PM) recruitment of the adaptor protein subunits. Graphs show quantifications of PM signal intensities from representative experiments, each data point represents a single root. TPLATE‐GFP mock: 49 ± 11 a.u. (mean ± SD), *n* = 16; β‐estradiol: 90 ± 24 a.u., *n* = 16. AP2A1‐TagRFP mock: 67 ± 6 a.u., n = 16; β‐estradiol: 98 ± 14 a.u., *n* = 16. Values were compared using *t*‐tests, ***, *P* ≤ 0.001. Bar, 10 μm, h, hours. (c) Total internal reflection fluorescence (TIRF) images of TPLATE‐GFP in roots of control and induced seedlings of *XVE»amiCHCa*. Silencing of *CHC* did not affect the density of TPLATE‐positive foci at the PM. Graph shows collated quantifications of average foci density from all experiments, each data point represents a single root. Mock: 159 ± 45 foci per 100 μm^2^ (mean ± SD), *n* = 20; β‐estradiol: 169 ± 31 foci per 100 μm^2^, *n* = 21. Values were compared using a *t*‐test, ns, not significant. Bar, 2 μm, h, hours.

In turn, after silencing of *CHC*, the intensity of both TPLATE‐GFP and AP2A1‐TagRFP marker signal was consistently increased at the PMs of RAM epidermis, as captured using CLSM (Fig. 3a,b). Signal levels of both adaptors were also increased at cell plates (Fig. S3A,B). The increased adaptor binding to the PMs and cell plates was not a result of a higher expression of the reporters, since silencing of *CHC* led to a decrease in *TPLATE‐GFP* and *AP2A1‐TagRFP* transcript levels (Fig. S4A). However, an evaluation of TPLATE‐GFP and AP2A1‐TagRFP protein levels in root tips by total signal measurement with epifluorescent microscopy revealed an increase in levels of both adaptors (Fig. S4B,C). Thus, the increased adaptor binding to the PMs and cell plates may be a result of their abnormal accumulation following *CHC* silencing. TIRF microscopy in early elongation zones of seedling roots did not reveal an increase in the average density of TPLATE‐GFP‐positive foci at the PM (Fig. 3c). The relatively weaker AP2A1‐TagRFP reporter was not analysed due to insufficient sensitivity of our TIRF microscopy setup. Thus, the effect observed with CLSM may be limited to the meristematic zone, or may represent an elevated adaptor recruitment into individual CCPs rather than an increase in the density of CCP initiation events.

In summary, silencing of *CHC* in *XVE»amiCHCa* lines affected all tested CME markers. The observations likely reflect CME inhibited at a stage after the initial recruitment of adaptor protein complexes. The variably observed increase in DRP1C signals at the PM might be interpreted as pools of dynamin recruited to CCPs from their initiation, rather than only at the time of scission (Fujimoto *et al*., 2010; Taylor *et al*., 2011; Gnyliukh *et al*., 2024), which stay or accumulate at the PMs similarly to the early‐arriving adaptor complexes.

### Effects of *CHC* silencing on secretory compartments

**Fig. 4:**
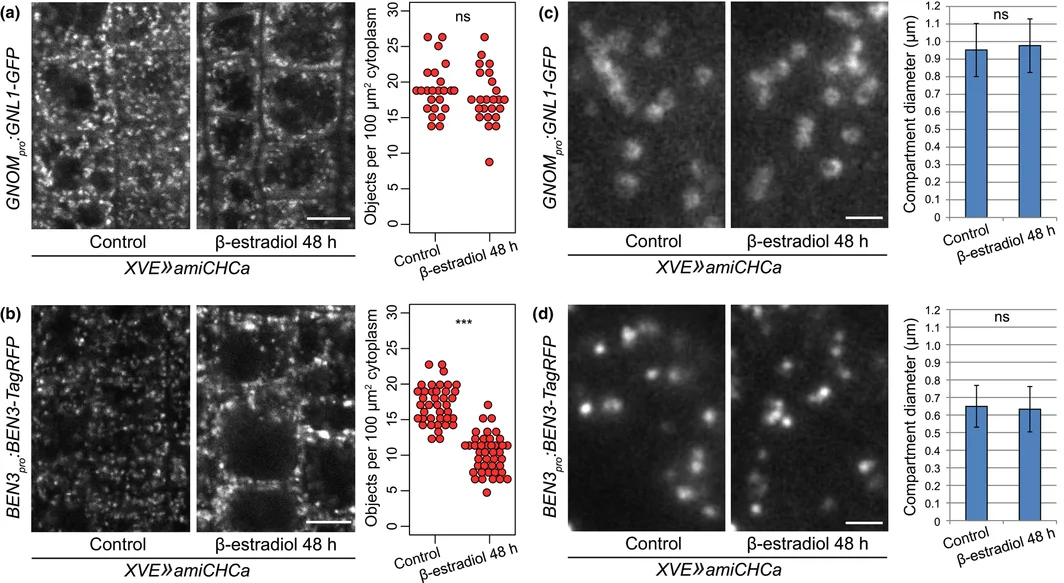
Effects of *CLATHRIN HEAVY CHAIN* (*CHC*) silencing in *Arabidopsis thaliana XVE»amiCHCa* on Golgi apparatus and *trans‐*Golgi Network/early endosome (TGN/EE). Confocal laser scanning microscopy (CLSM) images of GNL1‐GFP (a) and BEN3‐TagRFP (b) in root apical meristems (RAMs) of control and induced seedlings of *XVE»amiCHCa*. Bars, 10 μm, h, hours. Graphs show quantification of density of individual objects in cytoplasm, each data point representing one root. GNL1‐GFP control: 18.96 ± 3.49 objects per 100 μm^2^ (mean ± SD), *n* = 24; β‐estradiol: 17.86 ± 3.79 objects per 100 μm^2^, *n* = 24. BEN3‐TagRFP control: 17.16 ± 2.70 objects per 100 μm^2^, *n* = 39; β‐estradiol: 10.20 ± 2.69 objects per 100 μm^2^, *n* = 42. Values were compared using *t*‐tests, ***, *P* ≤ 0.001, ns, not significant. Total internal reflection fluorescence (TIRF) images of GNL1‐GFP (c) and BEN3‐TagRFP (d) in root early elongation zones of control and induced seedlings of *XVE»amiCHCa*. Bars, 2 μm, h, hours. Graphs show quantification of individual compartment diameters. GNL1‐GFP control: 0.95 ± 0.15 μm (mean ± SD), *n* = 180; β‐estradiol: 0.98 ± 0.15 μm, *n* = 210. BEN3‐TagRFP control: 0.65 ± 0.12 μm, *n* = 185; β‐estradiol: 0.63 ± 0.13 μm, *n* = 170. Values were compared using *t*‐tests, ns, not significant.

Next, we analysed the effect of *CHC* downregulation on GA and TGN/EE, organelles central to the activity of the secretory pathway. To observe the abundance and morphology of these endomembrane compartments, *XVE»amiCHCa* was transformed into lines expressing fluorescent protein fusions of GNOM‐LIKE1 (GNL1), an ARF‐GEF active at the GA (Richter *et al*., 2007; Teh & Moore, 2007) and BIG2/BEN3 (BFA‐VISUALISED TRAFFICKING DEFECTIVE3), a member of the BIG ARF‐GEF class acting at the TGN/EE (Richter *et al*., 2014; Kitakura *et al*., 2017). The fluorescent markers were analysed by CLSM in seedling RAM epidermis to enable observation of compartments on a cellular and tissue scale, and by TIRF microscopy in early elongation zones of seedling roots to enable imaging of individual organelles at high magnification. In induced *XVE»amiCHCa* lines, GNL1‐GFP labelled GA compartments of normal abundance and sizes (Fig. 4a,c). In turn, BEN3‐TagRFP signals observed with CLSM were fewer in number, with some objects unusually large and characterized by a high signal intensity (Fig. 4b). With the higher magnification enabled by TIRF imaging we observed that these larger signals are aggregates of multiple TGN/EEs (Fig. S5), while individual BEN3‐positive TGN/EE compartments have a normal appearance (Fig. 4d).

**Fig. 5:**
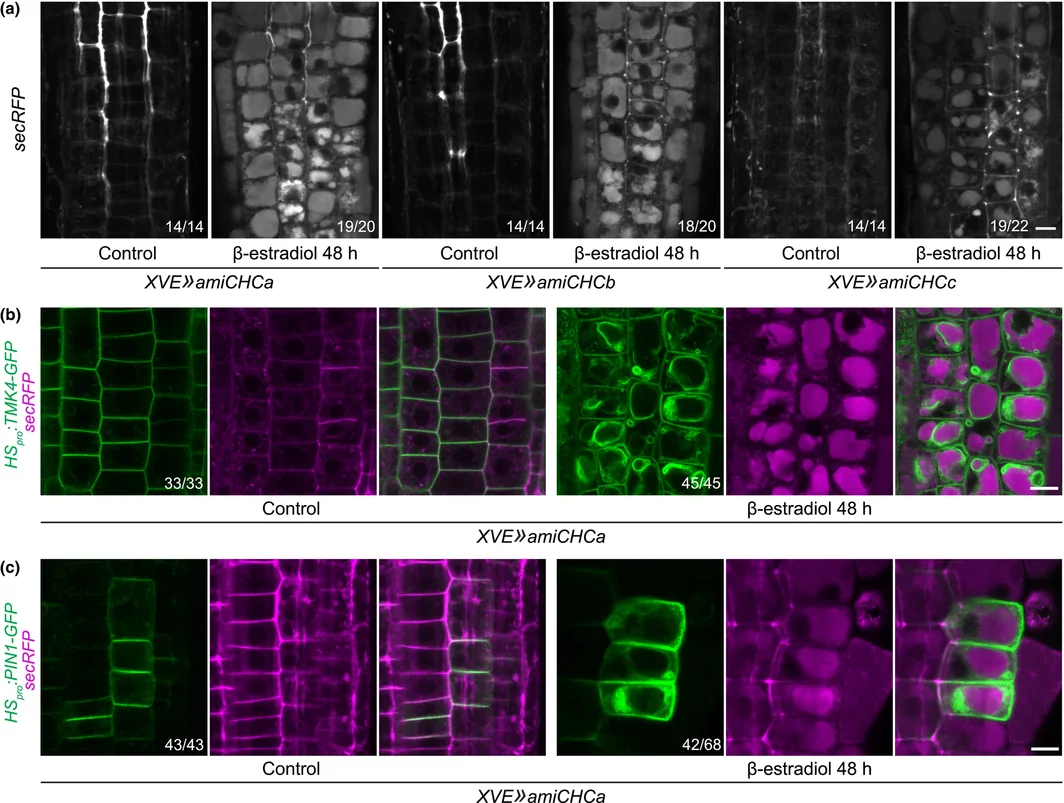
Effects of *CLATHRIN HEAVY CHAIN* (*CHC*) silencing on secRFP, TMK4‐GFP, and PIN1‐GFP in *Arabidopsis thaliana*. (a) Confocal laser scanning microscopy (CLSM) images of secRFP in root apical meristems (RAMs) of *XVE»amiCHCa*, *XVE»amiCHCb* and *XVE»amiCHCc* lines. Following *amiCHC* induction, large portions of secRFP are found relocated to the vacuole in all three lines. Numbers in bottom right corner indicate the ratio of roots with presented localizations. Bar, 10 μm, h, hours. (b) and (c). CLSM colocalization images of secRFP with TMK4‐GFP (b) and secRFP with PIN1‐GFP (c) in RAMs of *XVE»amiCHCa* lines. Following *amiCHC* induction, TMK4‐GFP and PIN1‐GFP are found partially localized to the tonoplast of the central, enlarged vacuole of *XVE»amiCHCa* RAM epidermal cells and more abundantly to the tonoplasts of smaller associated vacuoles. Numbers in bottom right corner indicate the ratio of roots with presented localizations, see text for details. Bars, 10 μm, h, hours.

Taken together, with the exception for a tendency of TGN/EE for aggregation, the appearance of internal compartments of the secretory pathway remains mostly normal in number and size following *CHC* silencing.

### Interference with secretory cargo traffic as a result of *CHC* downregulation

Finally, we characterized the effects of *CHC* downregulation on secretory cargoes. We employed the *secRFP XVE»amiCHCa*, *secRFP XVE»amiCHCb*, and *secRFP XVE»amiCHCc* lines, and additionally, we introduced heat‐shock‐inducible constructs expressing GFP fusions of PM‐targeted integral membrane proteins TRANS‐MEMBRANE KINASE 4 (TMK4; Dai *et al*., 2013; Wang *et al*., 2020) and PIN‐FORMED1 (PIN1; reviewed in Adamowski & Friml, 2015) into *secRFP XVE»amiCHCa*. In contrast to *HS*_*pro*_:*PIN1‐GFP*, *HS*_*pro*_:*TMK4‐GFP* construct expressed the TMK4‐GFP fusion protein at a lower level also without a heat‐shock induction (Fig. S6A), but we decided nevertheless to employ this line for experiments. By comparing peak PIN1‐GFP signal intensities between *PIN1*_*pro*_:*PIN1‐GFP* and *HS*_*pro*_:*PIN1‐GFP*, we confirmed that the heat‐shock‐inducible system leads to only modest expression (Fig. S6C). By applying heat shock and following over time the localization patterns of secRFP, GNL1‐GFP, and BEN3‐TagRFP, vacuolar morphology visualized with Neutral Red staining, and rates of endocytosis measured with the FM4‐64 endocytic tracer dye, we verified that heat shocks do not disrupt trafficking (Fig. S7A–E). Finally, we verified that the analysed cargoes traffic through the TGN/EE compartment, where clathrin is present, by applying Brefeldin A (BFA) to chemically inhibit trafficking processes dependent on BFA‐sensitive ARF‐GEFs, including post‐Golgi secretion (Naramoto *et al*., 2014; Richter *et al*., 2014). In these conditions, secRFP, TMK4‐GFP, and PIN1‐GFP became trapped in aggregates of TGN/EE compartments known as BFA bodies, where they colocalized with clathrin (Fig. S8A–C). Consistent with their transport in CCVs, TMK4 and PIN1 were found associated with purified plant CCVs (Dahhan *et al*., 2022).

When secRFP‐expressing lines were investigated by CLSM in seedling RAMs after *c*. 48 h of amiCHCa, amiCHCb, and amiCHCc induction, a large portion of secRFP was mislocalized to the vacuole in almost all seedlings analysed (Fig. 5a), instead of being secreted to the apoplast. This observation indicates a role of clathrin in secretion. We further analysed secRFP localization in *XVE»amiCHCa* at earlier points of amiRNA induction, when CHC protein levels remain at *c*. 80–90% of control (Fig. S1B). At 16 h of silencing, secRFP localization was normal in all seedlings analysed (Fig. S9A). The earliest visible effect of CHC silencing occurred at 24 h, when relocation of secRFP to vacuoles was observed in 34 of 44 seedlings analysed (Fig. S9A). At 32 h, 44 of 51 seedlings exhibited secRFP in vacuoles (Fig. S9A). Thus, interference with secretory traffic and a rerouting of secRFP to the vacuole is an early and sensitive consequence of clathrin silencing.

Similarly, both TMK4‐GFP and PIN1‐GFP were partially relocated from their normal PM localizations to the tonoplast of the main, enlarged vacuole of *XVE»amiCHCa* cells, as well as abundantly present at the membranes of smaller vacuoles often adjacent to the main vacuole (Fig. 5b,c). Of the two integral membrane protein markers, TMK4‐GFP appeared to undergo this tonoplast relocation more frequently. In seedlings imaged after *c*. 48 h of amiRNA induction and 7–8 h after reporter expression was first activated by heat shock, the abnormal localization pattern of TMK4‐GFP was observed in all of 45 RAMs imaged and essentially in all cells visible in the images, while PIN1‐GFP relocated to the tonoplast in 42 of 68 RAMs and regularly only in some of the cells expressing the marker. PIN1‐GFP localization at the tonoplast was observed in 8 of 48 RAMs imaged 3 h after reporter expression was first initiated (i.e. 2 h after 1 h heat‐shock induction), at which time the newly expressed PIN1‐GFP was often still transiting through intracellular compartments (Fig. S9B). Finally, like secRFP, both integral membrane proteins partially relocated to the tonoplast already at an earlier time point of 31 h of *CHC* silencing, with TMK4‐GFP weakly present in the tonoplast in all 12 seedlings analysed, and PIN1‐GFP in 3 of 12 seedlings (Fig. S9C,D).

Taken together, the results indicate a role of clathrin in late secretory transport of soluble and integral membrane protein cargoes. We envision that when a clathrin‐dependent secretory activity of the TGN becomes deficient, the cargoes are nonspecifically diverted towards the vacuole, most likely in multivesicular bodies (MVBs) maturating from TGNs (Scheuring *et al*., 2011). An alternative explanation of the observations, where the cargoes were first correctly delivered to the PM or the apoplast, then internalized by endocytosis, and then targeted to the vacuole, is unlikely for the following reasons. First, silencing of *CHC* strongly interferes with CME (Fig. 2). Second, TMK4 and PIN1 were abnormally delivered to the vacuolar membrane, and not the lumen, indicating that they lacked signals for the incorporation into intraluminal vesicles of the MVB by the endosomal sorting complexes required for transport (ESCRT) (Cullen & Steinberg, 2018; Rodriguez‐Furlan *et al*., 2019), as cargoes internalized from the PM and destined for the degradation in the vacuole do. Third, the alternative explanation would not be valid for secRFP, an artificial cargo that, to our knowledge, does not possess a PM receptor enabling its endocytosis. For similar reasons, we do not consider the observed relocation of secretory cargoes as a homeostatic mechanism, whereby the membrane and soluble cargoes are redirected towards the vacuole in a controlled fashion.

### Overexpression of AUXILIN‐LIKE1 inhibits CME preferentially over clathrin‐dependent late secretion

Overexpression of putative uncoating factors for endocytic CCVs, AUXILIN‐LIKE1/2, leads to inhibition of CME, as indicated by three lines of evidence: (1) inhibition of FM4‐64 endocytic tracer and protein cargo internalization, (2) changes in the PM recruitment and dynamics of proteins participating in CCV formation, and (3) an excess accumulation of membrane material at the cell periphery (Adamowski *et al*., 2018). This last observation was interpreted as resulting from a disruption of the normal balance between endo‐ and exocytosis, where continued exocytic delivery of new membrane in the absence of endocytosis gradually leads to a build‐up of membrane at the cell periphery. However, the effect of AUXILIN‐LIKE1/2 overexpression on late secretion was not explicitly addressed. Given the finding that silencing of *CHC* affects this transport step, we now assessed cargo secretion in *XVE»AUXILIN‐LIKE1*.

First, we generated a cross between *XVE»AUXILIN‐LIKE1* and *secRFP*. The *XVE»AUXILIN‐LIKE1* transgene became partially silenced in the cross, as evidenced by relatively weak seedling growth phenotypes compared with the original lines (Adamowski *et al*., 2018). Yet, following a 2‐d long induction by β‐estradiol, compared with the typical 1 d sufficient to observe the deleterious effects of AUXILIN‐LIKE1/2 overexpression (Adamowski *et al*., 2018), we identified some seedlings exhibiting clear AUXILIN‐LIKE1 overexpression phenotypes. In these seedlings, secRFP could be seen localized to the vacuole (Fig. S10A). In turn, the 2‐d long β‐estradiol treatment did not cause vacuolar relocation of secRFP in the wild‐type (Fig. S10B). The observation indicates that overexpression of AUXILIN‐LIKE1, like silencing of *CHC*, can affect secretion.

**Fig. 6:**
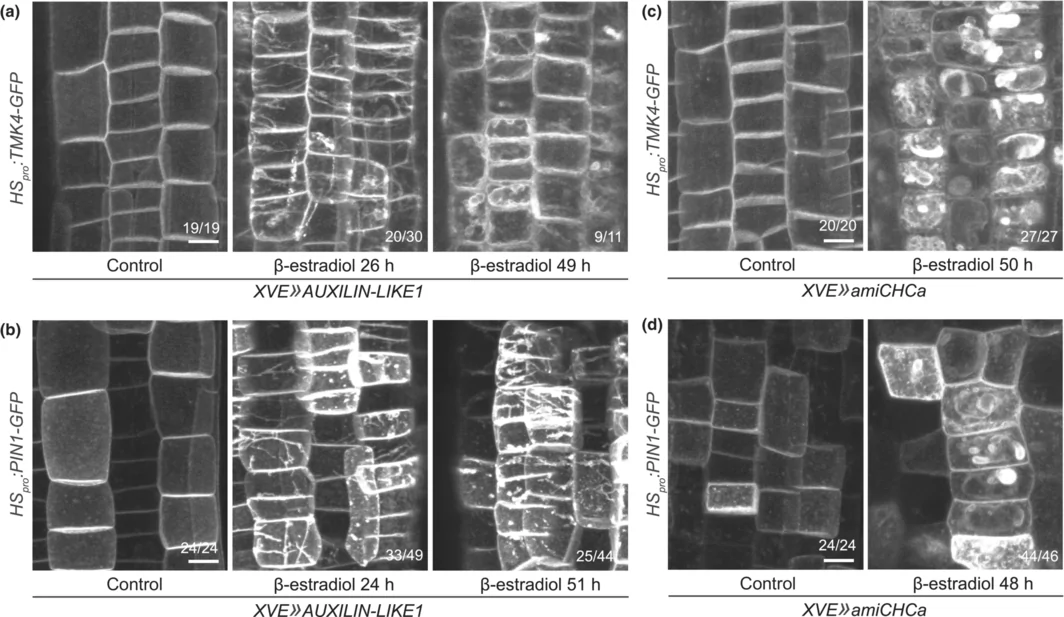
Comparison between AUXILIN‐LIKE1 overexpression and *CLATHRIN HEAVY CHAIN* (*CHC*) silencing effects on TMK4‐GFP and PIN1‐GFP trafficking and peripheral membrane deposition in *Arabidopsis thaliana*. Confocal laser scanning microscopy (CLSM) maximum projections of z‐stacks through root apical meristem (RAM) epidermal cell outer surfaces and interiors of *XVE»AUXILIN‐LIKE1* (a, b) and *XVE»amiCHCa* (c, d) expressing TMK4‐GFP (a, c) and PIN1‐GFP (b, d). Overexpression of AUXILIN‐LIKE1 for 24–26 h (hours) leads to deposition of excess filamentous membrane material at the plasma membrane (PM), as labelled by TMK4‐GFP and PIN1‐GFP. Overexpression of AUXILIN‐LIKE1 for 49–51 h interferes with TMK4‐GFP secretion and causes its relocation to the tonoplast, while PIN1‐GFP remains delivered to the PM in most seedlings analysed. Filamentous membrane deposits are not observed in *XVE»amiCHCa* lines induced for 48–50 h. Numbers in bottom right corner indicate the ratio of roots with presented localizations, see text for details. Bars, 10 μm.

For a more precise analysis, we turned to *XVE»AUXILIN‐LIKE1* lines transformed with *HS*_*pro*_:*TMK4‐GFP* and *HS*_*pro*_:*PIN1‐GFP*. In these lines, the AUXILIN‐LIKE1 overexpression phenotypes were normal. As in the case of *secRFP XVE»amiCHCa HS*_*pro*_:*TMK4‐GFP*, TMK4‐GFP expression was present also without heat‐shock induction (Fig. S6B). We first tested the effect of AUXILIN‐LIKE1 overexpression on TMK4‐GFP and PIN1‐GFP transport to the PM after 24–26 h of AUXILIN‐LIKE1 expression induction. At this point, the consequences of AUXILIN‐LIKE1 overexpression on CME are well visible (Adamowski *et al*., 2018), and in the analysed lines, they were evident by the abnormal morphology of the RAMs. Yet, in none of the 30 and 49 RAMs expressing TMK4‐GFP and PIN1‐GFP, respectively, were these cargoes relocated to the vacuole, indicating that unlike CME, secretion still functioned normally. Instead, in a majority of RAMs (TMK4‐GFP: 20 of 30 and PIN1‐GFP: 33 of 49), the PM pool of each protein was distributed in a form of thick filaments in at least part of the epidermal cells (Fig. 6a,b, middle), representing the excess accumulation of membrane material resulting from a continued secretory activity in the absence of CME. Note that these filaments are an accumulation of the membrane itself, rather than of the fluorescent protein fusion, as they were observed with the FM4‐64 membrane stain in a line not expressing any cargo reporter (Adamowski *et al*., 2018); consistently, TMK4‐GFP and PIN1‐GFP signals in the filaments colocalized with FM4‐64 (Fig. S11).

When assessed at a later time point of 49–51 h of AUXILIN‐LIKE1 expression, TMK4‐GFP relocated to the tonoplast in 9 of 11 RAMs (Fig. 6a, right), in some cases co‐existing with a localization in the PM filaments. Similar to the case of *CHC* silencing, PIN1‐GFP was less sensitive to the interference than TMK4‐GFP, and was still localized to membrane deposits at the PM like at the earlier time point in 25 of 44 RAMs (Fig. 6b, right), but tonoplast relocation was observed in 4 of 44 RAMs (Fig. S12A). Thus, although the strength of the effect differed between the two tested membrane protein cargoes, AUXILIN‐LIKE1 overexpression was able to affect their PM delivery at a later stage of induction, similarly as it interfered with secRFP secretion (Fig. S10A).

In summary, at early, but not later points of AUXILIN‐LIKE1 overexpression, inhibition of CME occurs without a concomitant inhibition of clathrin‐dependent late secretion. To complete the comparison between AUXILIN‐LIKE1 overexpression and *CHC* silencing, using confocal z‐stacks through outer faces of epidermal cells we verified if filamentous membrane deposits form in *XVE»amiCHCa* as in *XVE»AUXILIN‐LIKE1*. In contrast with *XVE»AUXILIN‐LIKE1*, after *c*. 48 h of *XVE»amiCHCa* expression, no membrane deposits were observed in 44 of 46 RAMs expressing PIN1‐GFP and 27 of 27 RAMs expressing TMK4‐GFP (Figs 6c,d, S12B). Earlier, at 31 h of *XVE»amiCHCa* induction, no membrane deposits were observed either (Fig. S9C,D). The near absence of these structures is consistent with the notion that unlike AUXILIN‐LIKE1 overexpression, *CHC* silencing inhibits endocytosis and secretion to a similar degree, not resulting in an imbalance between membrane addition and removal from the PM.

Taken together, overexpression of AUXILIN‐LIKE1, at least at earlier points of expression induction, preferentially inhibits endocytosis without having an equal effect on late secretion, as evidenced by the lack of interference with secretory cargo traffic and by the excess deposition of membranes at the cell periphery. While the exact mechanism by which overexpressed AUXILIN‐LIKE1 interferes with clathrin function is unknown, the overexpressed protein appears to preferentially target CCVs forming at the PM, but not TGN. This is consistent with the previously observed loss of PM pools of clathrin, but not the TGN pools of clathrin, following AUXILIN‐LIKE1 overexpression, and with the preferential PM, rather than TGN, localization of AUXILIN‐LIKE1/2 reporters (Adamowski *et al*., 2018). The observation that the two counteracting transport processes can be uncoupled in this line argues against the presence of a homeostatic mechanism that adjusts rates of secretion to the rates of endocytosis. The absence of such a mechanism, in turn, further supports our notion that the role of clathrin in late secretion detected in *amiCHC* lines (Fig. 5) is direct, since the observed effect of clathrin silencing on secretory cargoes cannot be interpreted merely as an indirect, homeostatic reaction of the secretory pathway to the inhibition of CME.

### Assessment of the rates of CME in secretory mutants

To explore further the possible homeostatic regulations at the PM interface, we asked the complementary question of whether endocytic rates react to the cellular rates of secretion. To address this, we assessed CME in mutants deficient in post‐Golgi secretion with CLSM and TIRF microscopy of CLC2 and TPLATE fluorescent reporters. We employed *ap1m2–1*, a mutant of the μ subunit of the AP‐1 clathrin adaptor (Park *et al*., 2013); *echidna* (*ech*), a mutant of a TGN‐localized secretory component likely associated with the Rab and ARF small GTPase machinery (Gendre *et al*., 2011, 2013; Jonsson *et al*., 2017; Ravikumar *et al*., 2018); and *sec5a‐2 sec5b‐1*, a mutant in the SEC5 subunit of the exocyst, a tethering complex responsible for the docking of SVs to the PM (Žárský *et al*., 2013; Zhu *et al*., 2018). All these mutants exhibit significant deficiencies characterized by a clear delay in growth and development in seedlings after 7 d of culture, when CLSM and TIRF imaging were conducted.

**Fig. 7:**
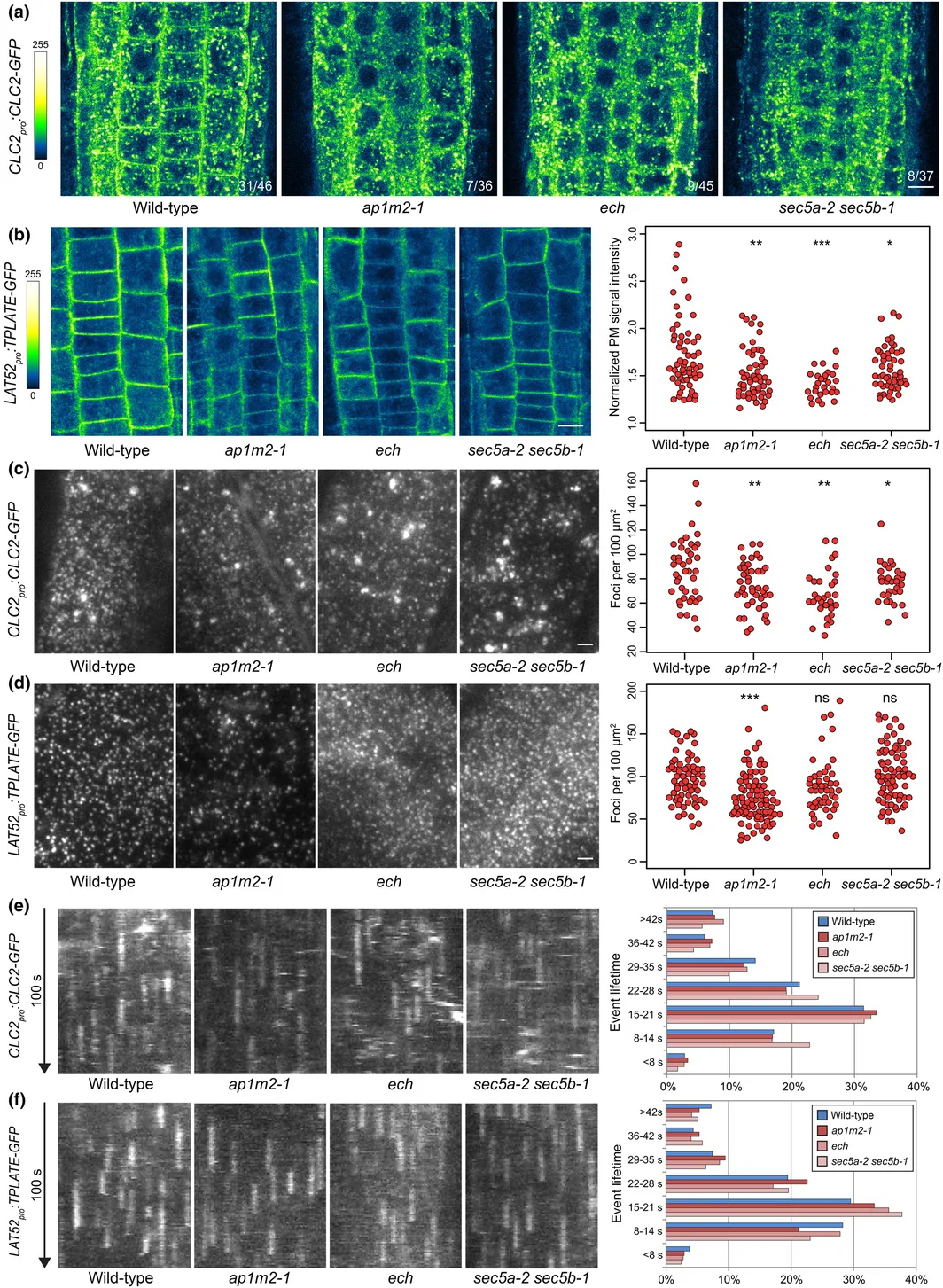
Characterization of clathrin‐mediated endocytosis (CME) in secretory mutants of *Arabidopsis thaliana*. (a) Confocal laser scanning microscopy (CLSM) images of CLC2‐GFP in root apical meristems (RAMs) of *ap1m2–1*, *ech*, and *sec5a‐2 sec5b‐1*. Numbers in bottom right corner indicate the ratio of roots with prevalent plasma membrane (PM) signals. Bar, 10 μm. (b) CLSM images of TPLATE‐GFP in RAMs of *ap1m2–1*, *ech*, and *sec5a‐2 sec5b‐1*. Bar, 10 μm. Graph shows quantification of PM signal intensity normalized to whole RAM signal levels, each data point represents one root. Wild‐type: 1.72 ± 0.39 (mean ± SD), *n* = 58; *ap1m2–1*: 1.53 ± 0.26, *n* = 52; *ech*: 1.41 ± 0.14, *n* = 28; *sec5a‐2 sec5b‐1*: 1.57 ± 0.22, *n* = 52. Mutant values were compared with the wild‐type using *t*‐tests, *, *P* ≤ 0.05; **, *P* ≤ 0.01; ***, *P* ≤ 0.001. (c, d) Total internal reflection fluorescence (TIRF) microscopic images and graphs visualizing foci densities of CLC2‐GFP (c) and TPLATE‐GFP (d) in seedling roots of *ap1m2–1*, *ech*, and *sec5a‐2 sec5b‐1*. Bars, 2 μm. Graphs show collated data from all experiments, each data point represents a measurement from a single cell. CLC2‐GFP signals at the *trans‐*Golgi Network (TGN) (large objects) were not included in the quantification. CLC2‐GFP wild‐type: 87 ± 25 foci per 100 μm^2^ (mean ± SD), *n* = 42; *ap1m2–1*: 74 ± 19 foci per 100 μm^2^, *n* = 45; *ech*: 68 ± 20 foci per 100 μm^2^, *n* = 30; *sec5a‐2 sec5b‐1*: 76 ± 15 foci per 100 μm^2^, *n* = 32. TPLATE‐GFP wild‐type: 97 ± 27 foci per 100 μm^2^, *n* = 68; *ap1m2–1*: 75 ± 29 foci per 100 μm^2^, *n* = 91; *ech*: 88 ± 33 foci per 100 μm^2^, *n* = 48; *sec5a‐2 sec5b‐1*: 103 ± 33 foci per 100 μm^2^, *n* = 73. Mutant values were compared with the wild‐type using *t*‐tests, *, *P* ≤ 0.05; **, *P* ≤ 0.01; ***, *P* ≤ 0.001; ns, not significant. (e, f) Kymographs of TIRF time lapses and graphs representing lifetime distributions of individual vesicle formation events visualized with CLC2‐GFP (e) and TPLATE‐GFP (f) in seedling roots of *ap1m2–1*, *ech*, and *sec5a‐2 sec5b‐1*. Between 849 and 876 individual events originating from four time lapses were measured per genotype and marker combination, s, seconds.

We first evaluated the state of CME in the mutants with CLSM of CLC2‐GFP and TPLATE‐GFP markers in seedling RAM epidermis. While CLC2‐GFP was clearly detectable at the PMs in 31 of 46 wild‐type seedlings, the majority of seedlings of all three mutants exhibited little or no PM signals of CLC2‐GFP (Fig. 7a). Similarly, the average intensity of TPLATE‐GFP signals at the PMs was decreased in all three mutants, although the effect was more pronounced in *ech* than in *ap1m2–1* and *sec5a‐2 sec5b‐1* (Fig. 7b).

Next, using TIRF microscopy, we quantified the density of CCPs labelled by CLC2‐GFP and by TPLATE‐GFP in the early elongation zone of the mutant seedling roots. In a total sample of 30 to 45 cells imaged in 18 to 20 seedlings of each genotype in the course of the experiments, we found a slight but significant reduction in the average density of CLC2‐GFP‐positive foci at the PMs of all three secretory mutants (Fig. 7c). In turn, in similar experiments with the TPLATE‐GFP marker, in a total sample of 48 to 91 cells taken from 18 to 34 seedlings of each genotype, we found only the *ap1m2–1* mutant to have a significant reduction in the density of TPLATE‐GFP foci, while *ech* and *sec5a‐2 sec5b‐1* had foci density not different from the control (Fig. 7d). Additionally, using both CLC2‐GFP and TPLATE‐GFP markers, we measured lifetimes of individual vesicle formation events. We found no systematic differences in lifetime distributions between the genotypes and concluded that individual CCVs formed normally in all three secretory mutants (Fig. 7e,f).

We further performed FM4‐64 endocytic tracer uptake experiments. Interestingly, in a sample of 39 to 50 seedlings per genotype, we detected an increase in intracellular FM4‐64 signals after 15 min of uptake in all three secretory mutants (Fig. S13). Rather than indicating increased endocytosis, we think this unanticipated observation is a result of the secretory defects in the mutants. While FM4‐64 is typically used to evaluate endocytosis, the dye is known to enter the secretory pathway as well (Bolte *et al*., 2004). We conceive that in the secretory mutants, FM4‐64 is internalized into TGN/EE at either a normal or at slightly reduced rate, but in contrast with the wild‐type, it is ineffectively returned to the PM by TGN‐to‐PM secretory traffic, leading to its higher net accumulation at TGN/EE.

Taken together, both the reduced occurrence of CLC2‐GFP at the PMs observed by CLSM (Fig. 7a) and the mildly reduced densities of CLC2‐GFP‐positive foci at the PMs observed by TIRF microscopy (Fig. 7c) indicate that secretory mutants form fewer CCPs at the PM, suggesting the presence of a weak homeostatic mechanism adjusting CME to secretory traffic. The impact on the TPLATE‐GFP marker was detected consistently only in *ap1m2–1*, but not in the other two secretory mutants, where the more sensitive TIRF method showed normal foci density (Fig. 7b,d), indicating that in contrast to clathrin recruitment, the recruitment of the TPLATE complex at the initiation of CCPs might not be regulated by the homeostatic mechanism.

## Discussion

In this work, we addressed two related questions about the function of the plant cell's endomembrane system: that of the role of TGN‐localized clathrin, and that of potential regulatory mechanisms mutually adjusting rates of endocytosis and secretion as part of a homeostatic control component within the endomembrane system.

### An involvement of clathrin in post‐Golgi secretion

The role of clathrin at the TGN has been a matter of much debate. Evidence obtained, especially with the use of mutants in components associated with clathrin in the vesicle formation process at the TGN, such as adaptors, support the involvement of CCVs in either vacuolar, or late secretory trafficking pathways (Feraru *et al*., 2010; Zwiewka *et al*., 2011; Park *et al*., 2013; Sauer *et al*., 2013; Robinson & Pimpl, 2014; Wang *et al*., 2014; Collins *et al*., 2020; Heinze *et al*., 2020; Shimizu *et al*., 2021; Dahhan *et al*., 2022; Liu *et al*., 2022). Our results from inducible downregulation of *CHC* expression support a function of clathrin in secretory transport of soluble and integral membrane protein cargo. The conclusions from *amiCHC* lines are specific to the degree that (1) the TGN retained presence and a normal appearance of individual compartments in TIRF microscopy, (2) the impact on secretory cargo transport was detected as an early and sensitive effect of even modest clathrin protein level reduction, and (3) the effect is not explained by an indirect, homeostatic response of the secretory pathway to inhibition of CME, since inhibition of CME does not trigger an arrest of secretion, as shown in the AUXILIN‐LIKE1 overexpression line. Yet, the role of clathrin in post‐Golgi secretory traffic need not be entirely direct, that is, the experiments do not constitute definitive evidence for the existence of CCVs forming at the TGN and destined for exocytosis. Clathrin at the TGN may support late secretion in a different way. One possibility is that beside a role in vesicle formation, clathrin possesses a structural function at the TGN needed to define a secretory domain. In another model, proposed in the mammalian field, AP‐1‐coated CCVs indirectly support secretion as carriers for retrograde traffic from post‐Golgi secretory compartments, back into TGN (Robinson *et al*., 2024). Following this model, the secretory role of plant AP‐1, and by extension, clathrin, is explained by the need for AP‐1 CCVs to retrieve SNAREs from mature TGN (Golgi‐independent TGN) back into Golgi for their normal function (Shimizu & Uemura, 2022; Robinson *et al*., 2024). On the contrary, in yeast, a class of ‘dense’ vesicles is known that are considered a type of SVs, since they accumulate in mutants deficient in exocytosis, for example in the exocyst mutant *sec6*, and contain some of the known secretory cargoes (Harsay & Bretscher, 1995; Mulholland *et al*., 1997). The formation of these SVs depends on clathrin and AP‐1 (Gall *et al*., 2002; Gurunathan *et al*., 2002; Harsay & Schekman, 2002) and they were observed with clathrin coats or in the process of uncoating (Gall *et al*., 2002). Thus, they may represent CCVs directly involved in late secretion. Yet, in light of the current understanding of the role of yeast AP‐1, these previously characterized vesicles might instead be interpreted as AP‐1‐coated CCVs responsible for retrograde transport within the Golgi (Casler *et al*., 2022), since those vesicles also carry secretory cargo (Casler *et al*., 2019). This alternative interpretation is consistent with the fact that dense SVs unexpectedly contain the Rab GTPase Ypt1 (Mulholland *et al*., 1997) known to act within the Golgi, but not in post‐Golgi secretion (Thomas *et al*., 2018). It is not clear, however, why should intra‐Golgi vesicles accumulate, rather than turn over normally, in mutants defective in exocytic components, such as the exocyst, as dense SVs do (Harsay & Bretscher, 1995; Mulholland *et al*., 1997), leaving this matter unresolved. Altogether, and returning to the role of TGN‐localized clathrin in plants, while our data clearly demonstrate its role in late secretion, the exact mechanism behind this function remains not fully understood.

### Homeostatic control of transport at the PM‐TGN/EE interface

Theoretical considerations and experiments (Riezman, 1985; Steer, 1988; Larson *et al*., 2017; Yan *et al*., 2021) suggest the existence of mechanisms within the endomembrane system that adjust the rates of individual trafficking processes to one another on a cellular scale. We scrutinized whether such homeostatic controls may exist between the rates of trafficking routes to and from the PM, post‐Golgi secretion and endocytosis, respectively. Through the analysis of lines overexpressing a putative uncoating factor for endocytic CCVs, AUXILIN‐LIKE1, where inhibition of endocytosis preceded any visible interference with secretory traffic, we conclude that a homeostatic mechanism modulating secretion to the current rates of endocytosis is unlikely to be present in plant cells. This conclusion appears intuitive when secretion is perceived as a fundamental cellular activity, required for functions as essential as the construction of the cell wall. In a complementary assessment of a potential mechanism adjusting endocytosis to the rates of secretion, by imaging of CME in secretory mutants, CLSM experiments consistently indicated a reduced recruitment of clathrin, and to a smaller degree, of the TPLATE complex, to the PM. Yet, with TIRF microscopy, which enables a more direct evaluation of CME due to the possibility of quantifying individual forming endocytic vesicles, we found a reduction in rates of CME using both these markers only in *ap1m2–1*, consistent with data reported by Yan *et al*. (2021), but in the other two secretory mutants, we found only CLC2, but not TPLATE‐positive foci, to be reduced. It may be speculated that *ap1m2–1* is deficient in secretion to a higher degree than the other mutants analysed, explaining why a decrease in CME also was more reliably detected. Currently, this possibility remains unexplored, and when all the gathered data are considered together, we conclude that endocytosis is adjusted to the rate of secretion by a weak mechanism that acts on clathrin recruitment to the PM, but might not act on the preceding recruitment of adaptors.

## Competing interests

None declared.

## Author contributions

MA, SSA and JF designed research and wrote the manuscript. MA, AG, IM, and SSA performed research. MA, AG, SSA and JF analysed data.

## Disclaimer

The New Phytologist Foundation remains neutral with regard to jurisdictional claims in maps and in any institutional affiliations.

## Supporting information


**Dataset S1** Original data associated with this study.

**Fig. S1** Expression analysis of *A. thaliana secRFP XVE»amiCHC* lines.
**Fig. S2** Morphology of *A. thaliana XVE»amiCHC* lines on control media.
**Fig. S3** Effects of *CHC* silencing in *A. thaliana XVE»amiCHCa* on cell plate localization of TPLATE‐GFP and AP2A1‐TagRFP.
**Fig. S4** Expression analysis of *TPLATE‐GFP* and *AP2A1‐TagRFP* in *A. thaliana XVE»amiCHCa*.
**Fig. S5** Aggregations of TGN/EE in *A. thaliana XVE»amiCHCa*.
**Fig. S6** Controls of heat‐shock‐inducible constructs in *A. thaliana*.
**Fig. S7** Controls of the heat‐shock induction system in *A. thaliana*.
**Fig. S8** Colocalization of cargo proteins with clathrin in *A. thaliana*.
**Fig. S9** Effects of CHC silencing in *A. thaliana* on secretory cargoes at early time points of amiRNA and cargo reporter expression.
**Fig. S10** Relocation of secRFP to the vacuole in *A. thaliana XVE»AUXILIN‐LIKE1*.
**Fig. S11** FM4‐64 staining of membrane filaments labelled with TMK4‐GFP and PIN1‐GFP in *A. thaliana XVE»AUXILIN‐LIKE1*.
**Fig. S12** Rarely observed cellular phenotypes in *A. thaliana XVE»AUXILIN‐LIKE1* and *XVE»amiCHCa* lines.
**Fig. S13** FM4‐64 uptake in secretory mutants of *A. thaliana*.
**Table S1** *Arabidopsis thaliana* lines generated as part of this study.
**Table S2** Primers used in this study.
**Table S3** Constructs generated as part of this study.

**Video S1** TIRF time lapse of CLC2‐GFP in control conditions.

**Video S2** TIRF time lapse of CLC2‐GFP in *XVE»amiCHCa* induced for 45 h.
Please note: Wiley is not responsible for the content or functionality of any Supporting Information supplied by the authors. Any queries (other than missing material) should be directed to the *New Phytologist* Central Office.

## Data Availability

Original data associated with this study have been deposited in Dataset S1. The accession nos. of *A. thaliana* genes used in this study are as follows: CHC1 (AT3G11130), CHC2 (AT3G08530), CLC2 (AT2G40060), TPLATE (AT3G01780), AP2A1 (AT5G22770), DRP1C (AT1G14830), GNOM‐LIKE1 (AT5G39500), BEN3/BIG2 (AT3G60860), TMK4 (AT3G23750), PIN1 (AT1G73590), AUXILIN‐LIKE1 (AT4G12780), AP1M2 (AT1G60780), ECHIDNA (AT1G09330), SEC5A (AT1G76850), SEC5B (AT1G21170), TUB2 (AT5G62690), and PP2AA3 (AT1G13320).
