# Supplementary material for: The role of clathrin in post‐Golgi secretion in plant cells
Source: New Phytol. 2026 Jul 20;251(6):3401–17. doi: 10.1111/nph.71454 (PMC13491243; doi:10.1111/nph.71454)
Supplement: Supplementary file 1 [file NPH-251-3401-s003.pdf]

New Phytologist Supporting Information

Article title: The role of clathrin in post-Golgi secretion in plant cells

Authors: Maciek Adamowski, Adam Gackowski, Ivana Matijević, Saqer S. Alotaibi, and Jiří Friml

Article acceptance date: 4 July 2026

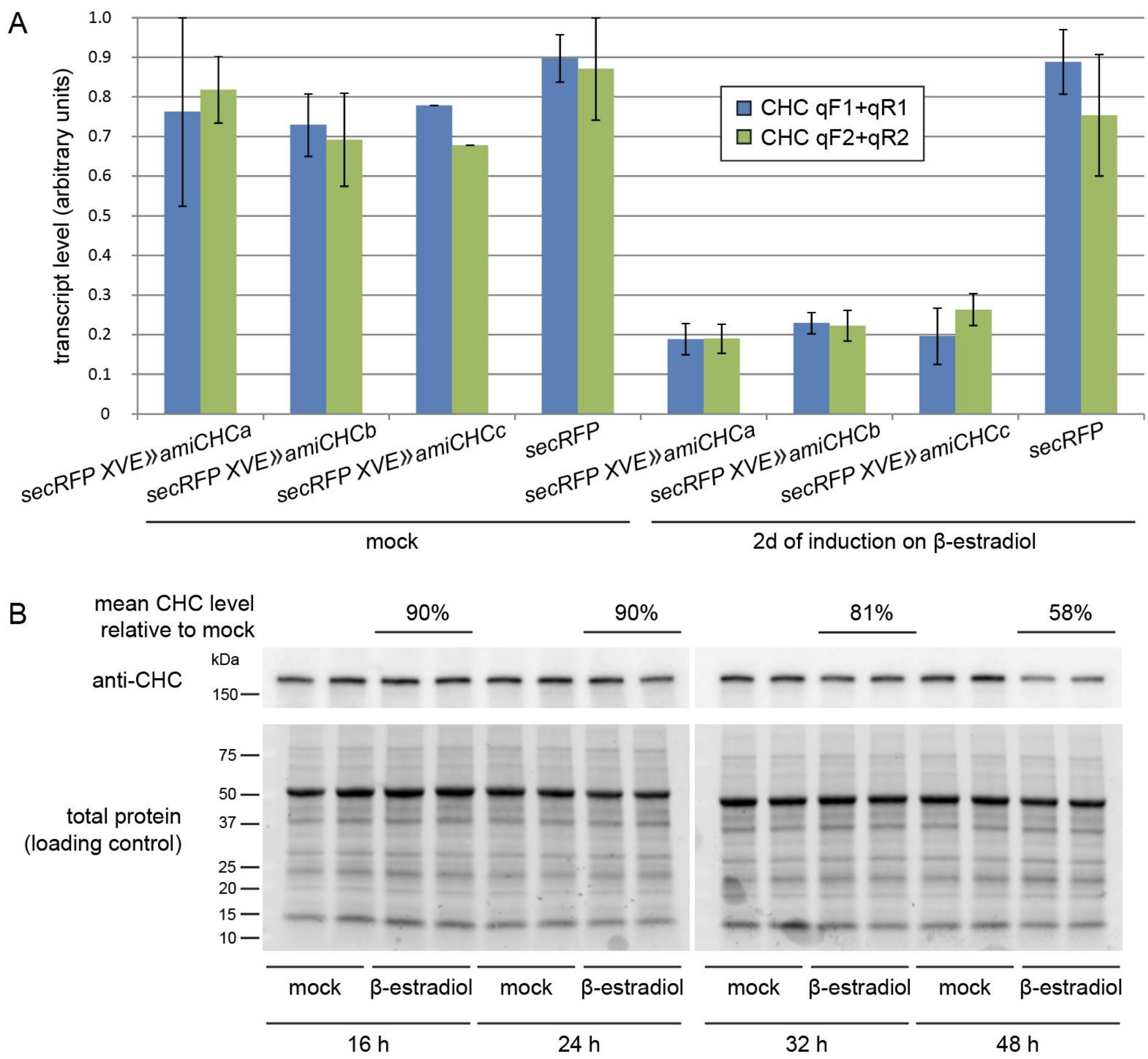

Suppl. Figure 1. Expression analysis of *A. thaliana secRFP XVE»amiCHC* lines

Related to Figure 1.

(A) Analysis of *CHC* transcript levels. The lines were induced on β-estradiol for 2 days (d) before RNA isolation. Transcript levels of *CHC1* and *CHC2* were assessed by qPCR with two primer pairs, each recognizing transcripts from both homologous genes, against reference genes *TUB2* and *PP2AA3*. Graphs represent mean ± SD from two biological replicates (seedling pools). Due to a technical error, 1 biological replicate was assessed for mock-treated *secRFP XVE»amiCHCc*.

(B) Analysis of CHC protein levels. *secRFP XVE»amiCHCa* was induced on β-estradiol and control media for 16 h (hours), 24 h, 32 h, and 48 h. CHC levels were evaluated by Western Blot using anti-CHC antibodies with total protein visualised by Stain-Free technology as loading control. The experiment was performed in duplicate, each lane shows one biological replicate (seedling pool). Quantification shows mean CHC level in induced samples relative to mock controls at a given time point. kDa – kilodalton.

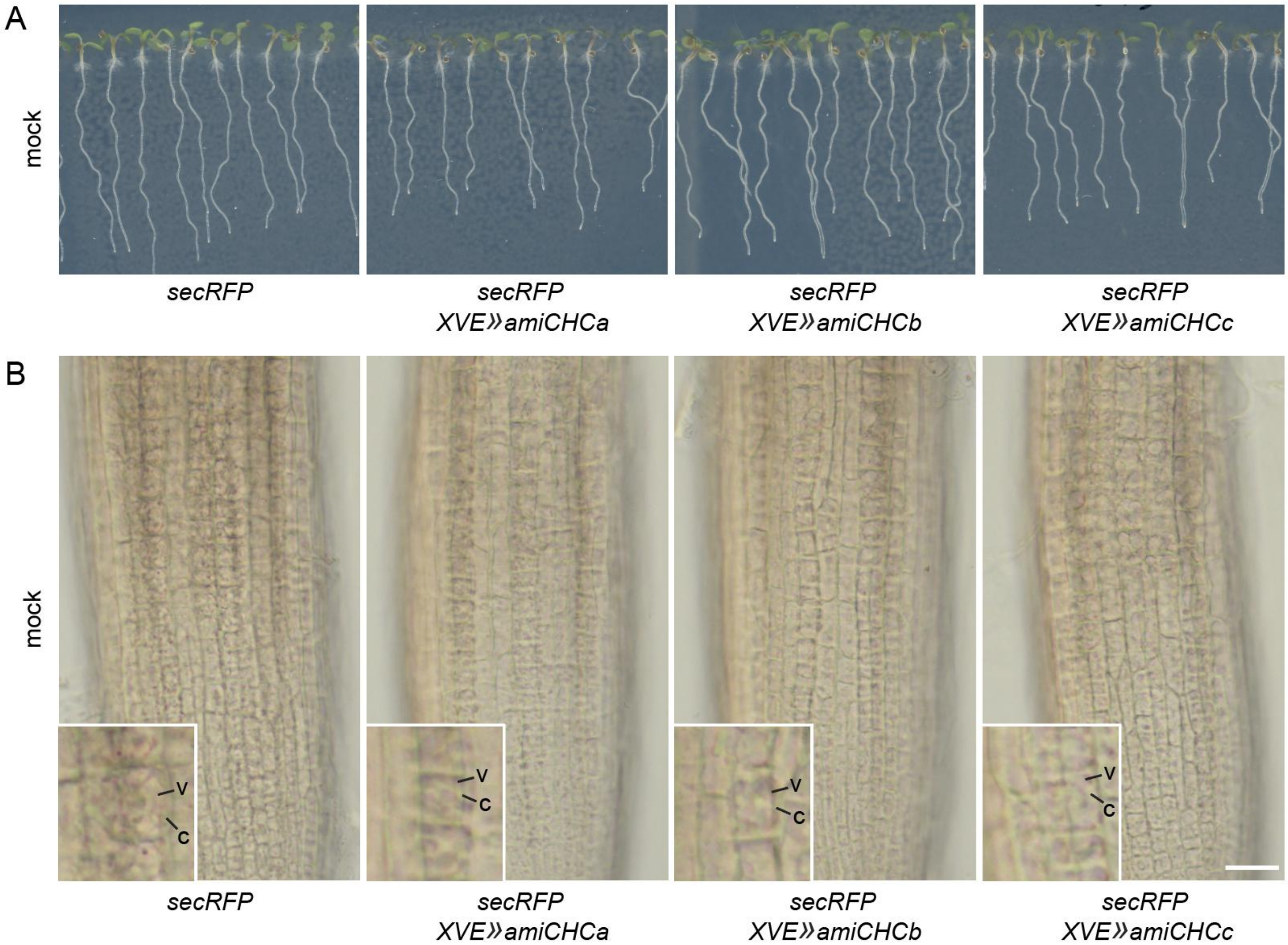

Suppl. Figure 2. Morphology of *A. thaliana XVE»amiCHC* lines on control media

Related to Figure 1.

(A) *secRFP XVE»amiCHC* lines grown on a control medium for 6 days.

(B) Microscopic images of RAMs of *secRFP XVE»amiCHC* lines from control media. Insets show magnified cells with fragmented vacuoles (v) and cytoplasm (c) indicated. Scale bar – 10 µm.

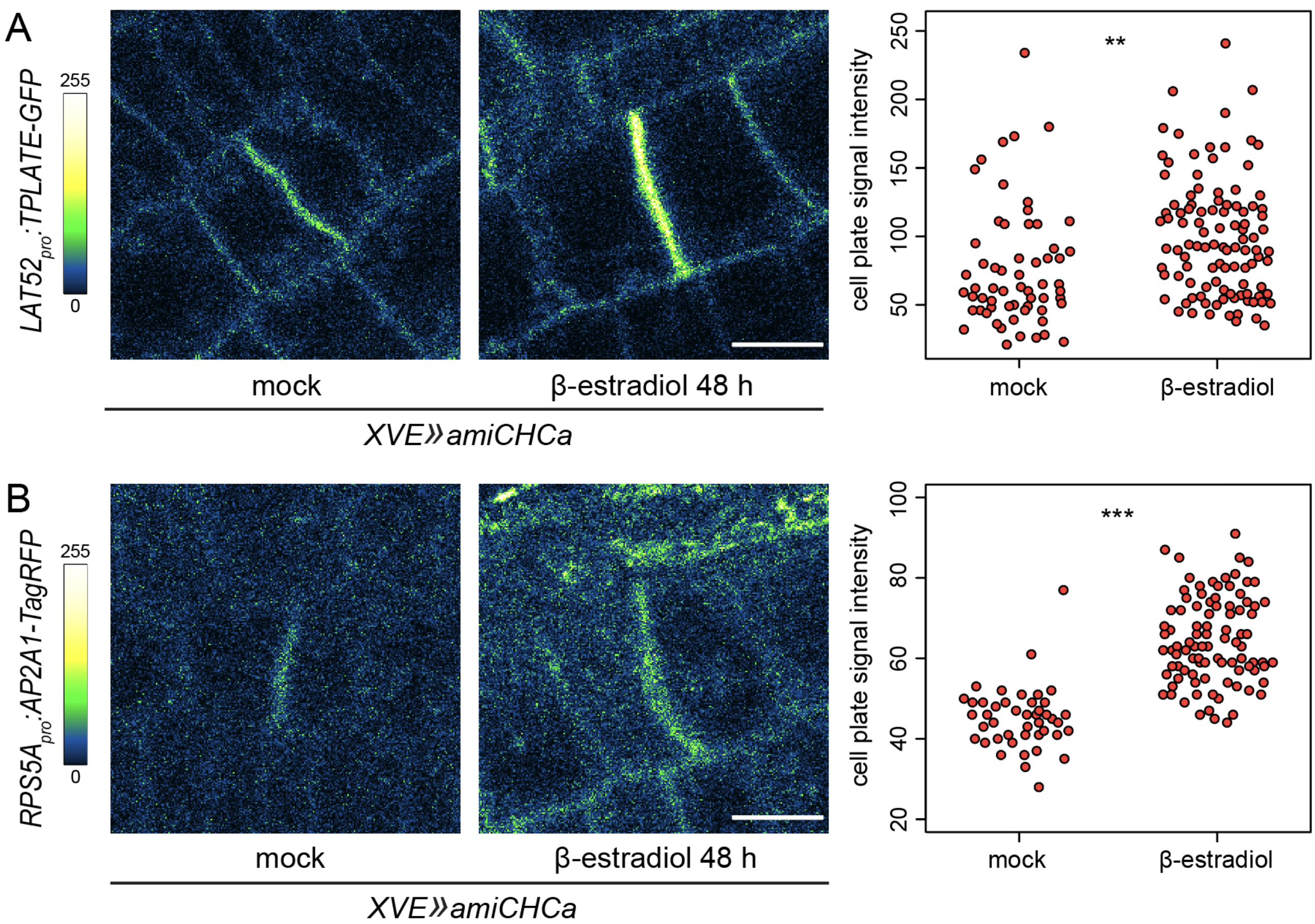

Suppl. Figure 3. Effects of *CHC* silencing in *A. thaliana XVE»amiCHCa* on cell plate localization of TPLATE-GFP and AP2A1-TagRFP

Related to Figure 3.

CLSM images of TPLATE-GFP (A) and AP2A1-TagRFP (B) in RAMs of control and induced seedlings of *XVE»amiCHCa*. Graphs show quantifications of cell plate signal intensities from a representative experiment, each data point represents a single cell plate. TPLATE-GFP mock: 76 ± 43 a.u. (mean ± s.d.), n=61; β-estradiol: 99 ± 43 a.u., n=106. AP2A1-TagRFP mock: 45 ± 8 a.u., n=44; β-estradiol: 65 ± 11 a.u., n=94. Values were compared using *t* tests, ** P≤0.01, *** P≤0.001. Scale bars – 10 µm, h – hours.

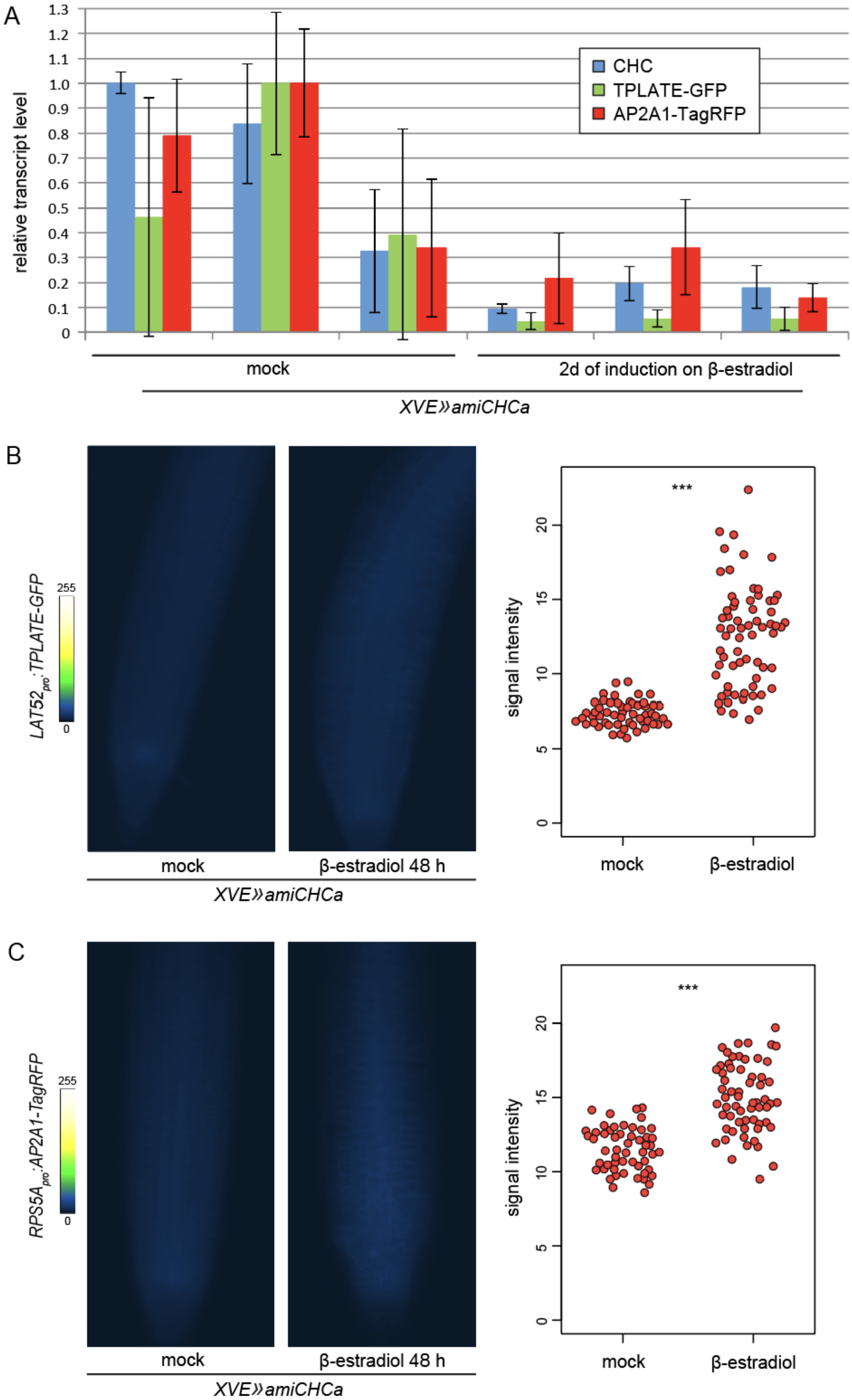

Suppl. Figure 4. Expression analysis of *TPLATE-GFP* and *AP2A1-TagRFP* in *A. thaliana XVE»amiCHCa*

Related to Figure 3.

(A) Analysis of *TPLATE-GFP and AP2A1-TagRFP* transcript levels by qPCR. *XVE»amiCHCa* line was induced on β-estradiol for 2 days (d) before RNA isolation. Transcript levels of *CHC1* and

CHC2, TPLATE-GFP, and *AP2A1-TagRFP* were assessed against reference genes *TUB2* and *PP2AA3* in three biological replicates (seedling pools) per condition shown as three sets of graphs. Graphs represent mean ± SD of three technical replicates.

(B, C) Evaluation of TPLATE-GFP (B) and AP2A1-TagRFP (C) protein levels in RAMs by epifluorescent microscopy. Graphs show quantifications of signal intensities, each data point represents a single root. TPLATE-GFP mock: 7.34 ± 0.83 a.u. (mean ± s.d.), n=55; β-estradiol: 12.54 ± 3.40 a.u., n=66. AP2A1-TagRFP mock: 11.50 ± 1.47 a.u., n=54; β-estradiol: 14.98 ± 2.32 a.u., n=62. Values were compared using *t* tests, *** P≤0.001. h – hours.

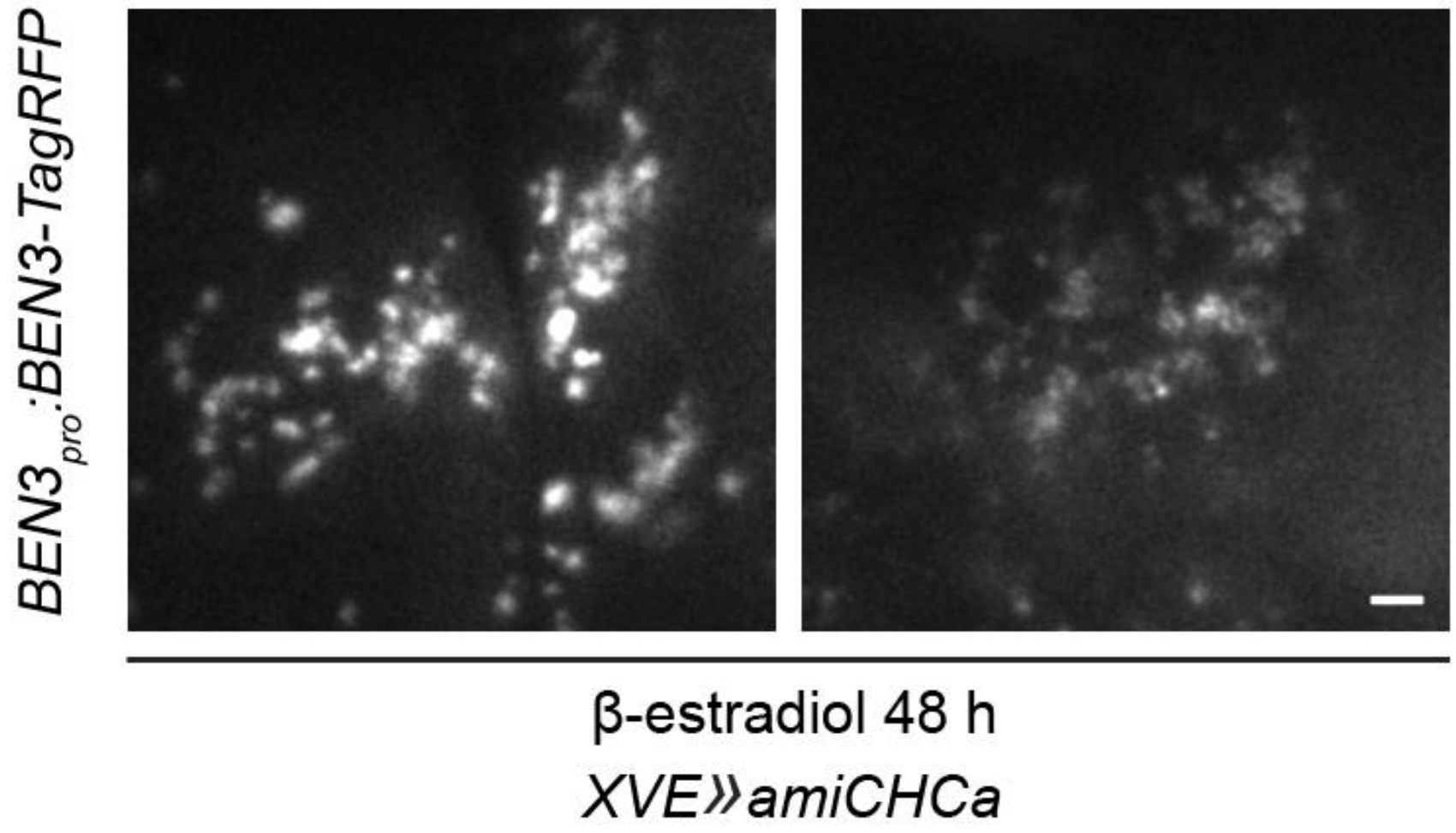

Suppl. Figure 5. Aggregations of TGN/EE in *A. thaliana XVE»amiCHCa*

Related to Figure 4.

TIRF microscopic images of BEN3-TagRFP showing aggregations of individual TGN/EE compartments observed in induced *XVE»amiCHCa* line. Scale bar – 2 µm, h – hours.

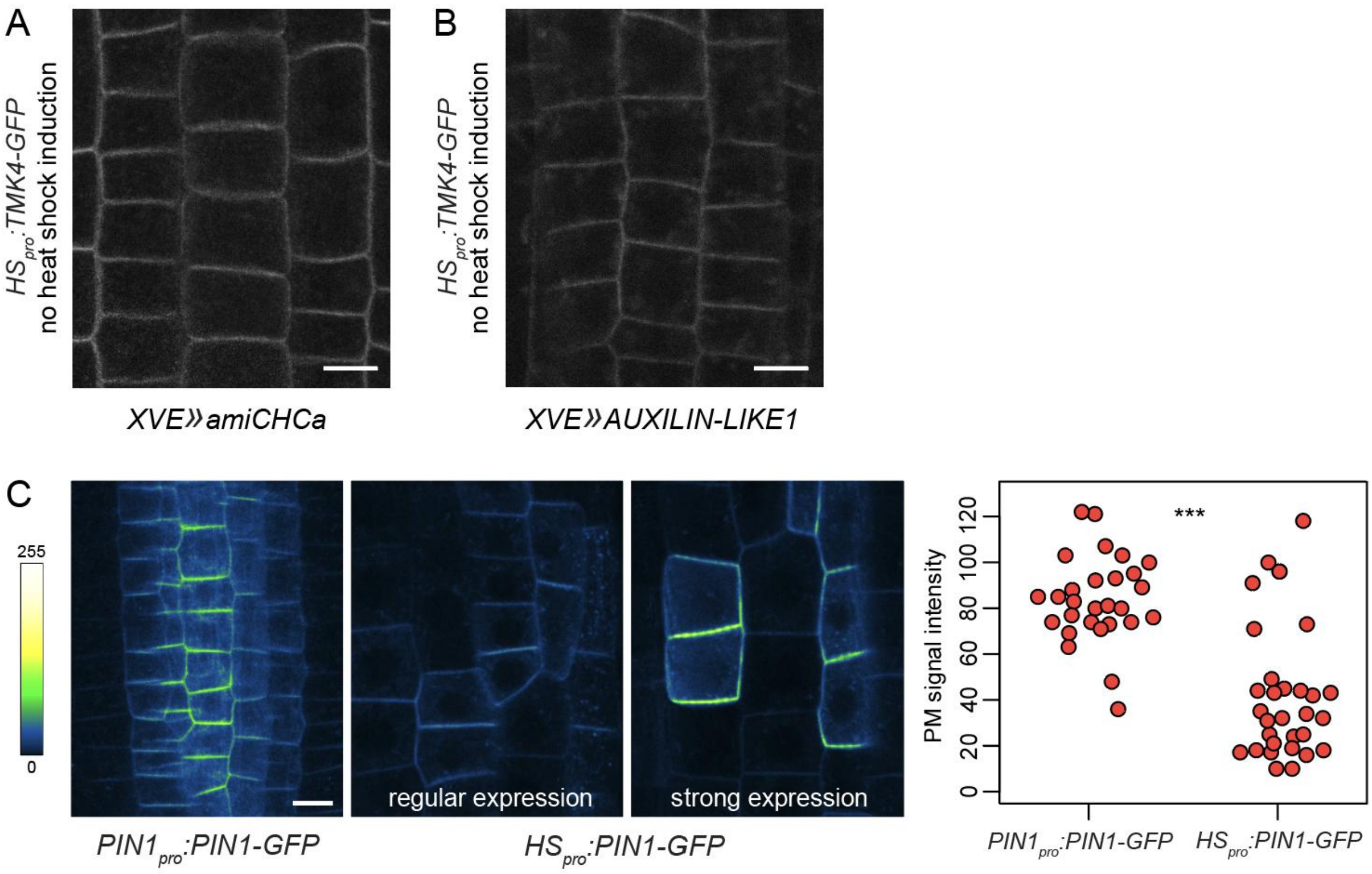

Suppl. Figure 6. Controls of heat shock-inducible constructs in *A. thaliana*

Related to Figures 5 and 6.

(A, B) CLSM images showing expression of TMK4-GFP in *XVE»amiCHCa* (A) and *XVE»AUXILIN-LIKE1* (B) without heat shock induction of the transgene. Scale bars – 10 µm.

(C) CLSM images of PIN1-GFP driven by the native promoter and by the heat shock promoter. Graph shows quantification of PM signals collated from two experiment repetitions, each data point represents a single root. The majority of roots expressing PIN1-GFP from the heat shock promoter were expressing at levels lower than the native promoter, while few were expressing at levels comparable to the native promoter. To compare peak expression levels between the lines, cells with highest expression observed in each root were quantified. *$PIN1_{pro}$:PIN1-GFP*: 84 ± 19 a.u. (mean ± s.d.), n=28; *HS_{pro}:PIN1-GFP*: 43 ± 30 a.u., n=31. Values were compared using a *t* test, *** P≤0.001. Scale bar – 10 µm.

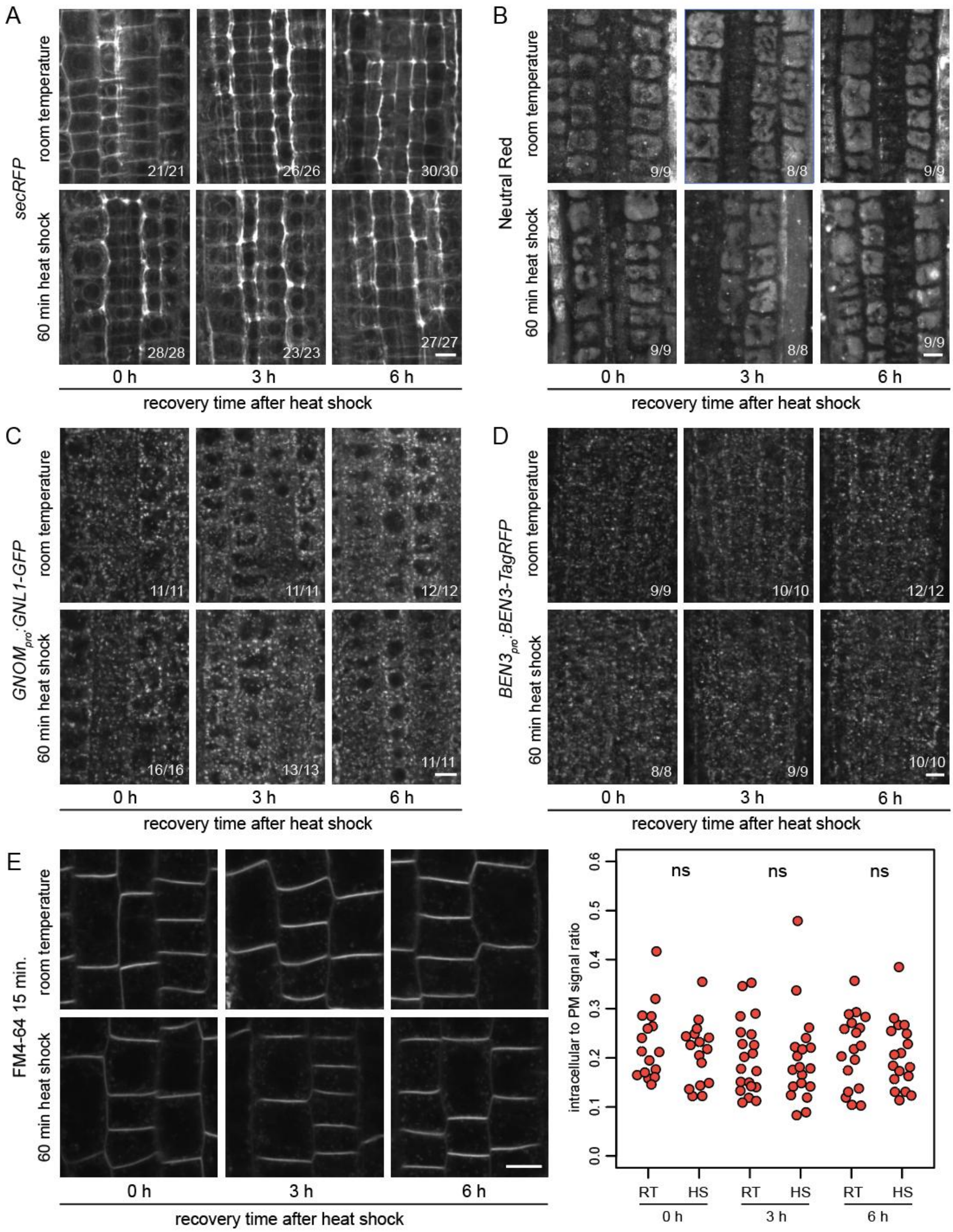

Suppl. Figure 7. Controls of the heat shock induction system in *A. thaliana*

Related to Figure 5.

(A-D) CLSM images of secRFP (A), Neutral Red staining (B), GNL1-GFP (C), and BEN3-TagRFP (D) after 0 h (hours), 3 h and 6 h of recovery from a heat shock treatment along with room

temperature controls. Numbers in bottom right corner indicate the ratio of roots with (A) normal secRFP localization pattern, (B) normal vacuole morphology, (C) normal cellular distribution of Golgi apparatus, and (D) normal cellular distribution of TGNs. Scale bars – 10 µm.

(E) CLSM images of FM4-64 after 15 minutes (min.) of uptake into RAM epidermal cells in the wild type after 0 h, 3 h and 6 h of recovery from a heat shock treatment along with room temperature controls. Scale bar – 10 µm. Graph shows quantification of intracellular to PM signal ratio, each data point represents one root. RT – room temperature, HS – heat shock. RT 0 h: 0.23 ± 0.07 (mean ± s.d.), n=16; HS 0 h: 0.21 ± 0.06, n=16; RT 3 h: 0.20 ± 0.07, n=20; HS 3 h: 0.20 ± 0.09, n=19; RT 6 h: 0.22 ± 0.07, n=18; HS 6 h: 0.21 ± 0.07, n=18. HS values were compared with RT at each time point using *t* tests, ns – not significant.

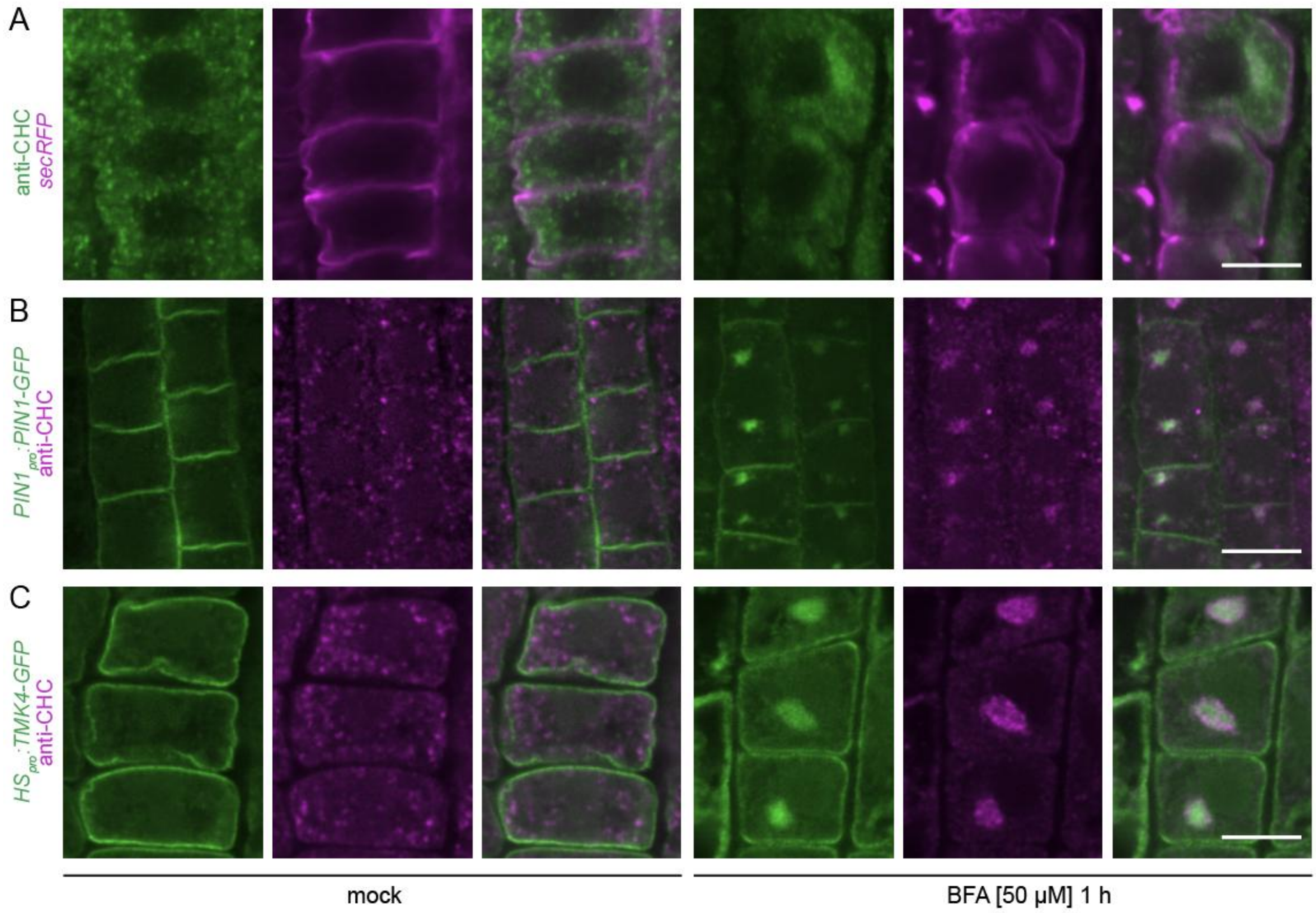

Suppl. Figure 8. Colocalisation of cargo proteins with clathrin in *A. thaliana*

Related to Figure 5.

CLSM images of CHC immunostaining in colocalisation with secRFP (A), PIN1-GFP (B), and TMK4-GFP (C) in control conditions and after 1 h (hour) of BFA treatment. Scale bars – 10 µm.

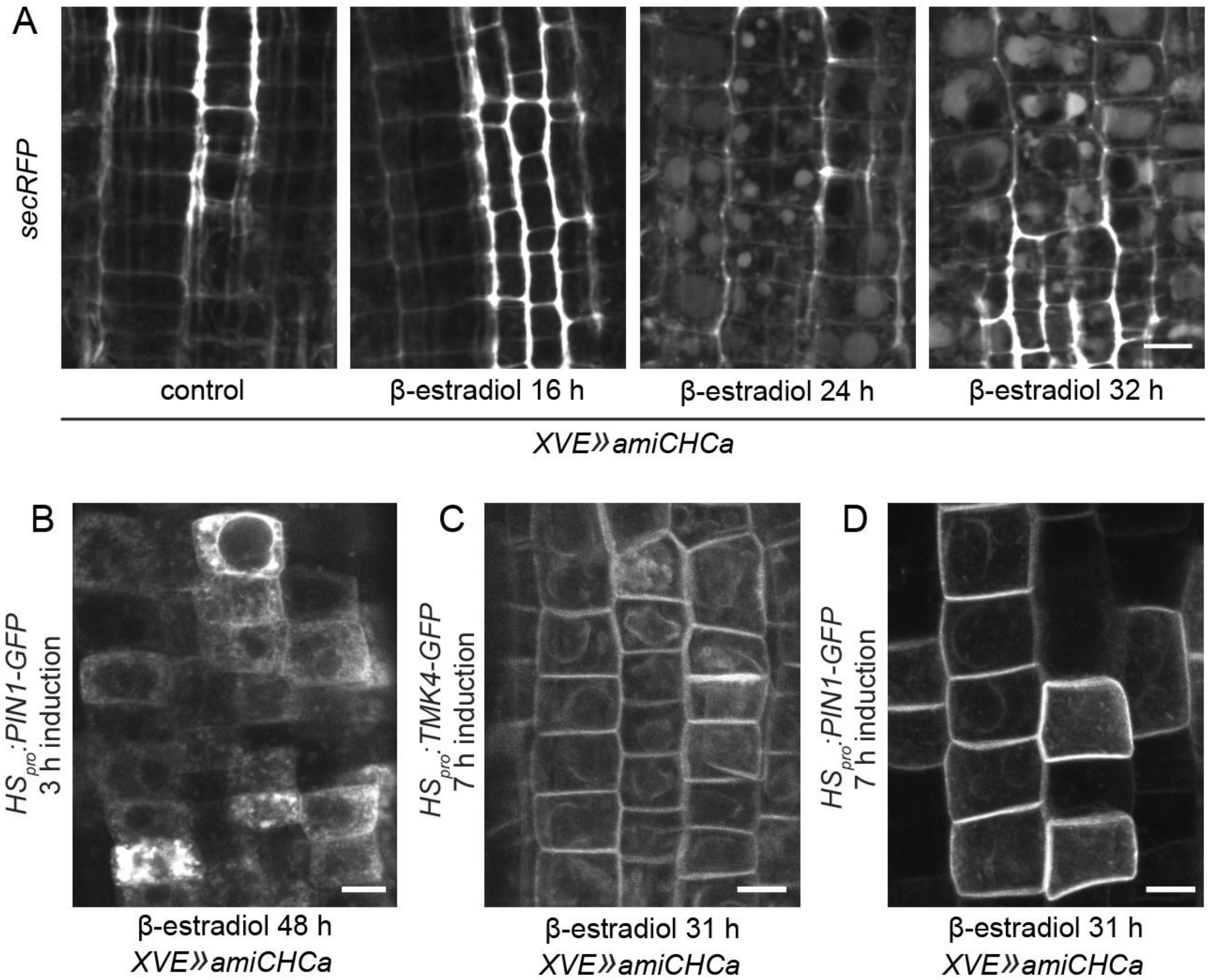

Suppl. Figure 9. Effects of CHC silencing in *A. thaliana* on secretory cargoes at early time points of amiRNA and cargo reporter expression

Related to Figure 5.

(A) CLSM images of secRFP in RAMs of *XVE»amiCHCa* at early time points of amiRNA induction. Scale bar – 10 μm, h – hours.

(B) CLSM image of PIN1-GFP 3 h (hours) after reporter expression activation by heat shock in *XVE»amiCHCa* induced for 48 h. Scale bar – 10 μm.

(C) and (D). Maximum projections of 9 μm z-stacks of CLSM images of TMK4-GFP (C) and PIN1-GFP (D) in RAMs of *XVE»amiCHCa* after 31 h (hours) of amiRNA induction. Scale bars – 10 μm.

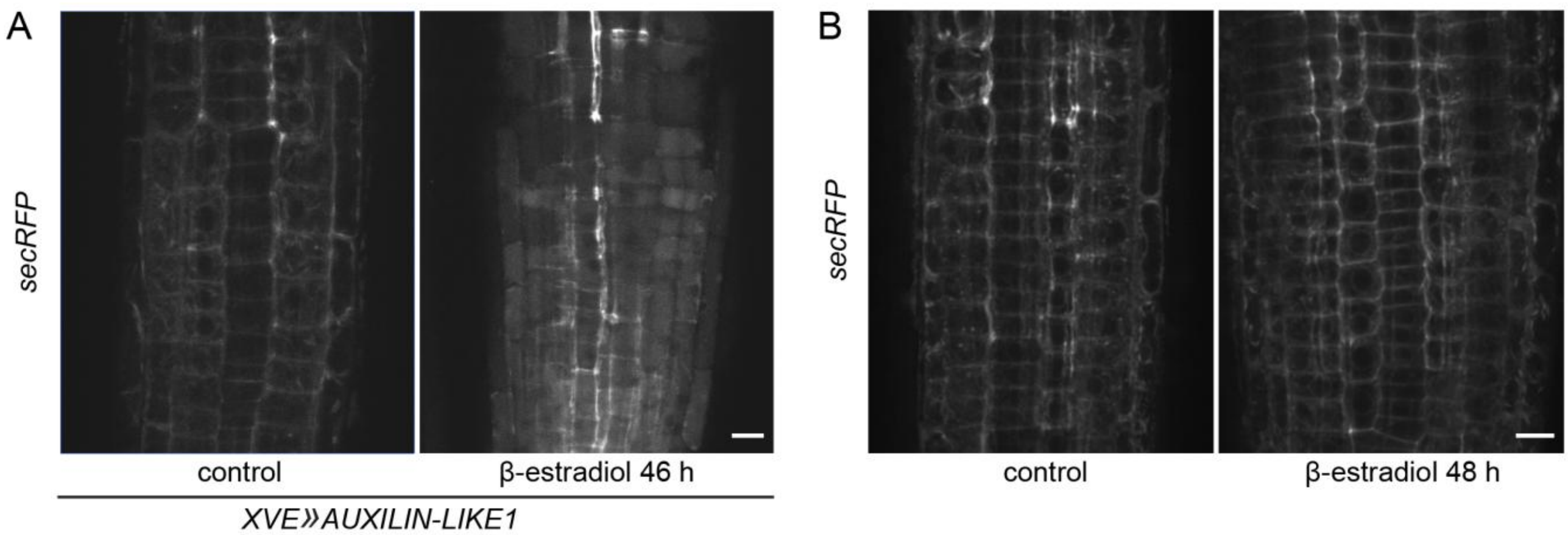

Suppl. Figure 10. Relocation of secRFP to the vacuole in *A. thaliana XVE»AUXILIN-LIKE1*

Related to Figure 6.

(A) CLSM images of secRFP in RAMs of *XVE»AUXILIN-LIKE1* line. In seedlings exhibiting AUXILIN-LIKE1 overexpression phenotypes in this cross, secRFP is found partially localized to the vacuole after 46 h (hours) of induction. Scale bar – 10 μm.

(B) CLSM images of secRFP in RAMs of the wild type. β-estradiol treatment does not interfere with secRFP trafficking. Scale bar – 10 μm, h – hours.

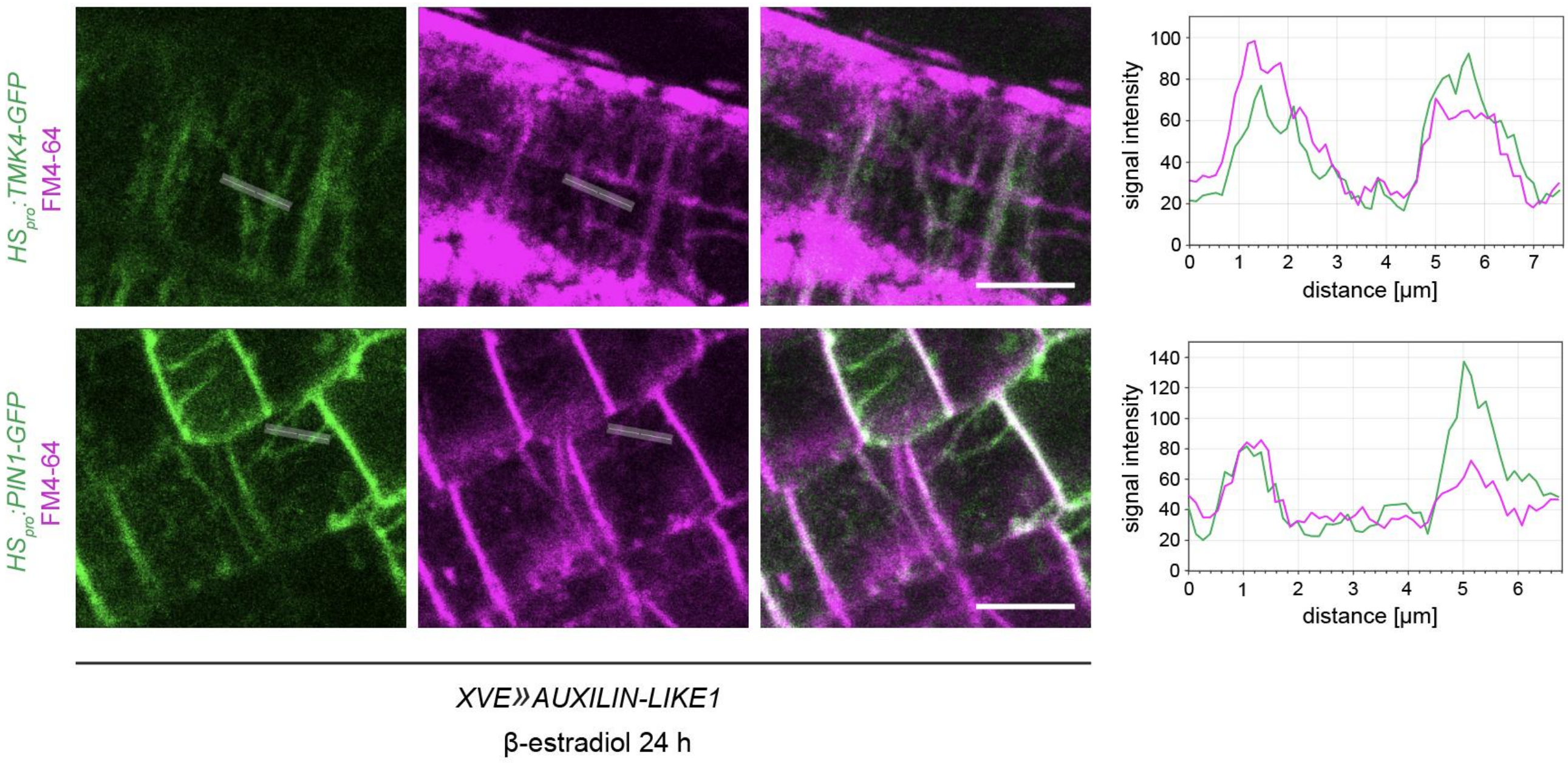

Suppl. Figure 11. FM4-64 staining of membrane filaments labelled with TMK4-GFP and PIN1-GFP in *A. thaliana XVE»AUXILIN-LIKE1*

Related to Figure 6.

CLSM images of TMK4-GFP and PIN1-GFP in colocalisation with FM4-64 in *XVE»AUXILIN-LIKE1* line. The fluorescent reporters localize to membrane filaments that stain with FM4-64. Scale bars – 10 µm. Graphs show quantification of GFP and FM4-64 signal intensities along lines indicated on CLSM images. h – hours.

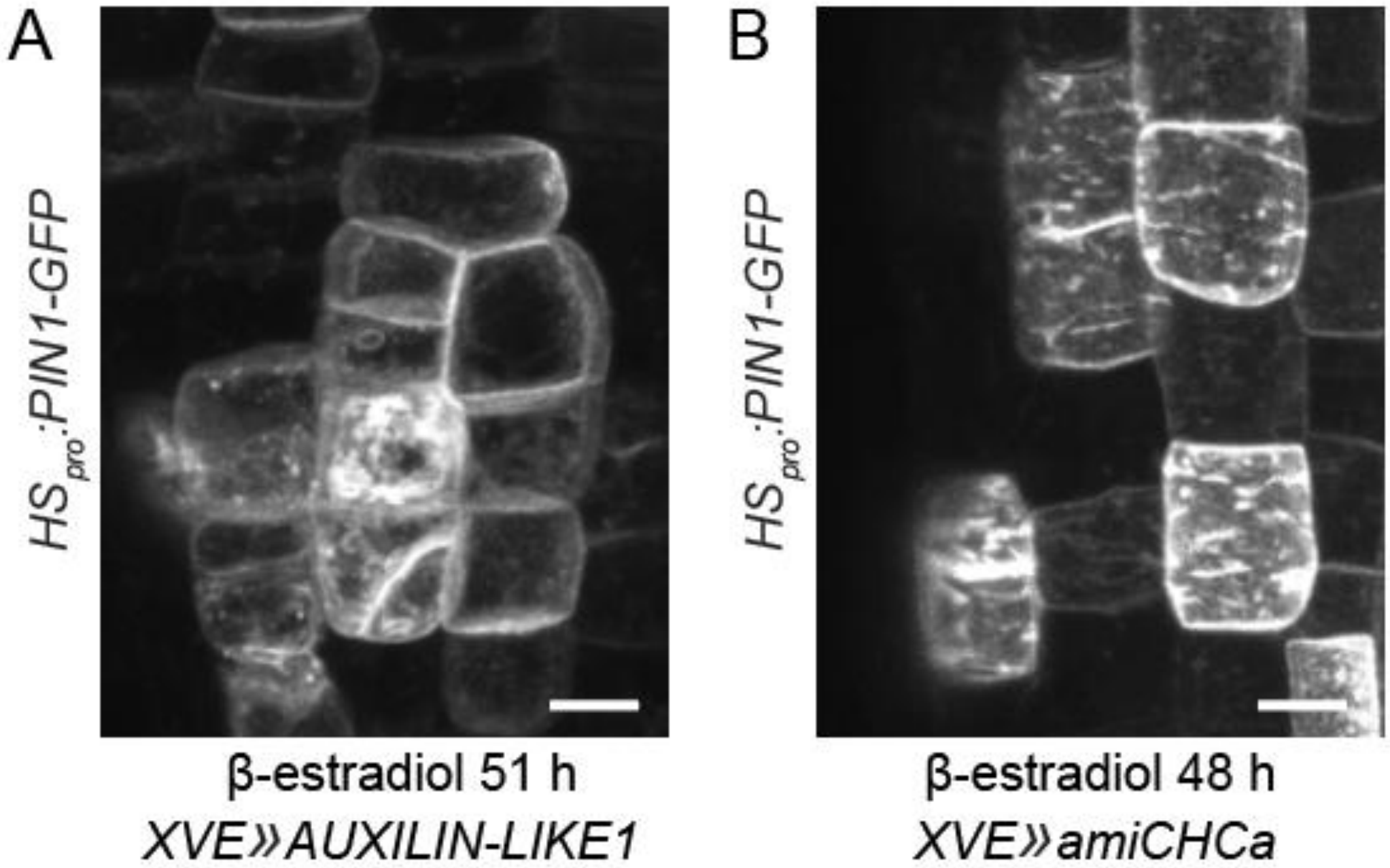

Suppl. Figure 12. Rarely observed cellular phenotypes in *A. thaliana XVE»AUXILIN-LIKE1* and *XVE»amiCHCa* lines

Related to Figure 6.

Maximum projections of 9 µm z-stacks of CLSM images. Scale bars – 10 µm.

(A) Rare localization of PIN1-GFP to the vacuole following AUXILIN-LIKE1 overexpression for 51 h (hours).

(B) Rare presence of filamentous membrane deposits labelled with PIN1-GFP in *XVE»amiCHCa* line induced for 48 h (hours).

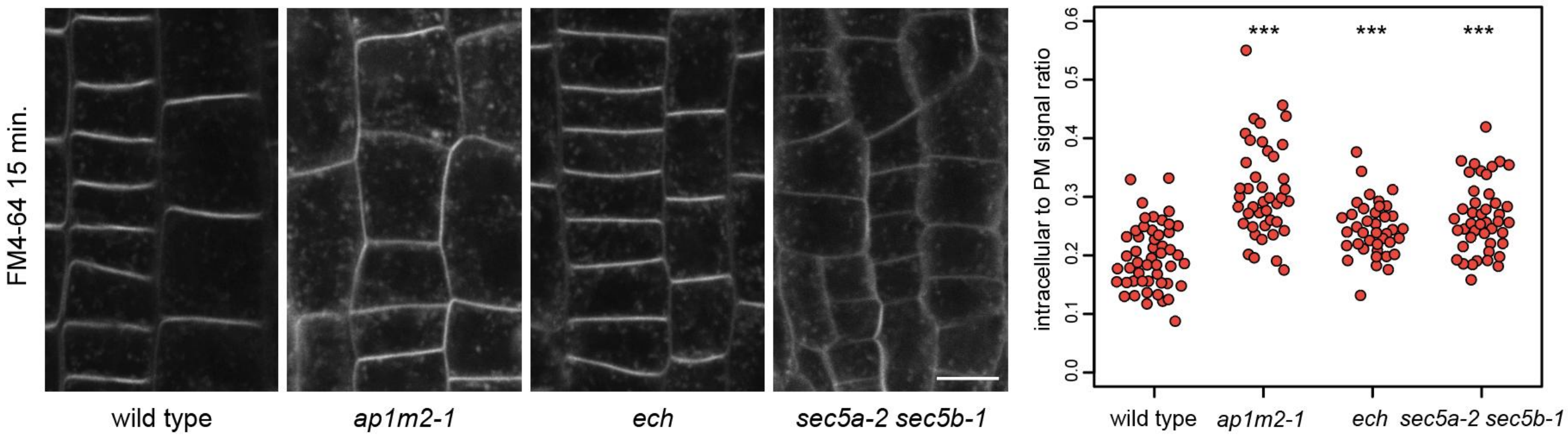

Suppl. Figure 13. FM4-64 uptake in secretory mutants of *A. thaliana*

Related to Figure 7.

CLSM images of FM4-64 after 15 minutes (min.) of uptake into RAM epidermal cells in wild type and *ap1m2-1, ech,* and *sec5a-2 sec5b-1*. Scale bar – 10 µm. Graph shows quantification of intracellular to PM signal ratio, each data point represents one root. Wild type: 0.20 ± 0.05 (mean ± s.d.), n=50; *ap1m2-1*: 0.31 ± 0.08, n=42; *ech*: 0.25 ± 0.05, n=39; *sec5a-2 sec5b-1*: 0.26 ± 0.06, n=44. Mutant values were compared with the wild type using *t* tests, *** P≤0.001.

Suppl. Table 1. *A. thaliana* lines generated as part of this study

Line name	Notes
secRFP XVE»amiCHCa	
secRFP XVE»amiCHCb	
secRFP XVE»amiCHCc	
$CLC2_{pro}$:CLC2-GFP $UBQ10_{pro}$:mCh-AUXILIN-LIKE1 XVE»amiCHCa	
$LAT52_{pro}$:TPLATE-GFP $RPS5A_{pro}$:AP2A1-TagRFP tplate XVE»amiCHCa	
$DRP1C_{pro}$:DRP1C-GFP XVE»amiCHCa	
$BEN3_{pro}$:BEN3-TagRFP XVE»amiCHCa	
$GNOM_{pro}$:GNOM-LIKE1-GFP XVE»amiCHCa	
secRFP HS_{pro}:PIN1-GFP XVE»amiCHCa	
secRFP HS_{pro}:TMK4-GFP XVE»amiCHCa	
secRFP XVE»AUXILIN-LIKE1	
HS_{pro}:PIN1-GFP XVE»AUXILIN-LIKE1	
HS_{pro}:TMK4-GFP XVE»AUXILIN-LIKE1	
ech $CLC2_{pro}$:CLC2-GFP	SAIL_163_E09
ech $LAT52_{pro}$:TPLATE-GFP $RPS5A_{pro}$:AP2A1-TagRFP	SAIL_163_E09
sec5a-2 sec5b-1 $CLC2_{pro}$:CLC2-GFP	GK-731C01; SALK_001525
sec5a-2 sec5b-1 $LAT52_{pro}$:TPLATE-GFP $RPS5A_{pro}$:AP2A1-TagRFP	GK-731C01; SALK_001525
ap1m2-1 $CLC2_{pro}$:CLC2-GFP	FLAG_293C11
ap1m2-1 $LAT52_{pro}$:TPLATE-GFP $RPS5A_{pro}$:AP2A1-TagRFP	FLAG_293C11

Suppl. Table 2. Primers used in this study

Primer name	Sequence	Purpose
ech-F	acagccattgtcgtcctctt	*ech* genotyping
ech-R	TCATTGATCTCGTTCCACCA	
ap1m2-1-F	tttggtgctggcagTATGAG	*ap1m2-1* genotyping
ap1m2-1-R	cagaaaaggaaagtggcaca	
sec5a-2-F	AGGAAGATGATGGTGCTTGG	*sec5a-2* genotyping
sec5a-2-R	CAAATCTGCTGCAGTCGTGT	
sec5b-1-F	ctctttggttgtggcagTCA	*sec5b-1* genotyping
sec5b-1-R	TTTGCCAACTCTCTCGCTTT	
LB-FLAG	CTACAAATTGCCTTTTCTTATCGAC	
LBb1.3	ATTTTGCCGATTTCGGAAC	
LB-SAIL-3	TAGCATCTGAATTTCATAACCAATCTCGATACAC	
LB-GK_o8409	ATATTGACCATCATACTCATTGC	
CHCa-I	gaTCTCGCAGTACTTACCCACAAtctctctttttgtattcc	amiCHCa cloning
CHCa-II	gaTTGTGGGTAAGTACTGCGAGAtcaaagagaatcaatga	
CHCa-III	gaTTATGGGTAAGTAGTGCGAGTtcacaggtcgtgatatg	
CHCa-IV	gaACTCGCACTACTTACCCATAAtctacatatatattcct	
CHCb-I	gaTGCAAAATTTACTGCTCCCTGtctctctttttgtattcc	amiCHCb cloning
CHCb-II	gaCAGGGAGCAGTAAATTTTGCAtcaaagagaatcaatga	
CHCb-III	gaCAAGGAGCAGTAATTTTTGCTtcacaggtcgtgatatg	
CHCb-IV	gaAGCAAAAATTACTGCTCCTTGtctacatatatattcct	
CHCc-I	gaTTATTTGACCAGTCTTGGCAGtctctctttttgtattcc	amiCHCc cloning
CHCc-II	gaCTGCCAAGACTGGTCAAATAAtcaaagagaatcaatga	
CHCc-III	gaCTACCAAGACTGGACAAATATtcacaggtcgtgatatg	
CHCc-IV	gaATATTTGTCCAGTCTTGGTAGtctacatatatattcct	
attB1-amiR-F	GGGGACAAGTTTGTACAAAAAAGCAGGCTCCCCAAACACACGCTCGGA	amiCHC cloning
attB2-amiR-R	GGGGACCACTTTGTACAAGAAAGCTGGGTCCCCATGGCGATGCCTTAAA	
attB1-TMK4-F	GGGGACAAGTTTGTACAAAAAAGCAGGCTCAATGGAGGCTCCTAC	TMK4 cloning
attB2-TMK4-Rns	GGGGACCACTTTGTACAAGAAAGCTGGGTCCCGACCATCAGCTGA	

attB1-PIN1-F	ggggacaagtttgtacaaaaaagcaggctcgatgattacggcggcggacttctaccac	PIN1-GFP cloning
attB2-PIN1-R	ggggaccactttgtacaagaaagctgggtctcatagacccaagagaatgtagtagag	
TUB2-qF	AAACTCACTACCCCCAGCTTTG	qPCR
TUB2-qR	CACCAGACATAGTAGCAGAAATCAAGT	qPCR
PP2AA3-qF	TAACGTGGCCAAAATGATGC	qPCR
PP2AA3-qR	GTTCTCCACAACCGCTTGGT	qPCR
CHC-qF1	GTTCAGGCTTGCAAGGAGTA	qPCR
CHC-qR1	TCAGGATCCTCACTCATACTCAA	qPCR
CHC-qF2	CCGTATCTCCTCCAGTTTATCC	qPCR
CHC-qR2	ACCTCCCATTCCTGGCATC	qPCR
GFP-qF	ATCATGGCCGACAAGCAGAA	qPCR
GFP-qR	CTCGATGTTGTGGCGGATCT	qPCR
TagRFP-qF	TCAGAGGGGTGAACTTCCCA	qPCR
TagRFP-qR	CTCCCAGCCGAGTGTTTTCT	qPCR

Suppl. Table 3. Constructs generated as part of this study

Plasmid name	Notes
PIN1-GFP-2/pDONR221	with STOP codon
TMK4/pDONR221	without STOP codon
amiRNA-CHCa/pDONR221	
amiRNA-CHCb/pDONR221	
amiRNA-CHCc/pDONR221	
proHS/pDONRP4P1r	
UBQ10-XVE»amiRNA-CHCa/pH7m24GW,3	
UBQ10-XVE»amiRNA-CHCa/pK7m24GW,3	
UBQ10-XVE»amiRNA-CHCa/pB7m24GW,3	
UBQ10-XVE»amiRNA-CHCb/pB7m24GW,3	
UBQ10-XVE»amiRNA-CHCc/pB7m24GW,3	
HS:PIN1-GFP-2/pK7m24GW,3	
HS:TMK4-GFP/pK7m34GW	

Suppl. Video 1. TIRF time lapse of CLC2-GFP in control conditions

TIRF time lapse of CLC2-GFP in a root of non-induced *XVE»amiCHCa* seedling. Time measurement is given in mm:ss (m – minutes, s – seconds) format.

Suppl. Video 2. TIRF time lapse of CLC2-GFP in XVE»amiCHCa induced for 45 h

TIRF time lapse of CLC2-GFP in a root of *XVE»amiCHCa* seedling induced for 45 hours. Time measurement is given in mm:ss (m – minutes, s – seconds) format.
